# ANGPTL2‐mediated epigenetic repression of MHC‐I in tumor cells accelerates tumor immune evasion

**DOI:** 10.1002/1878-0261.13490

**Published:** 2023-08-07

**Authors:** Tsuyoshi Kadomatsu, Chiaki Hara, Ryoma Kurahashi, Haruki Horiguchi, Jun Morinaga, Keishi Miyata, Sohtaro Kurano, Hisashi Kanemaru, Satoshi Fukushima, Kimi Araki, Masaya Baba, W. Marston Linehan, Tomomi Kamba, Yuichi Oike

**Affiliations:** ^1^ Department of Molecular Genetics, Graduate School of Medical Sciences Kumamoto University Japan; ^2^ Center for Metabolic Regulation of Healthy Aging (CMHA), Graduate School of Medical Sciences Kumamoto University Japan; ^3^ Department of Urology, Graduate School of Medical Sciences Kumamoto University Japan; ^4^ Department of Aging and Geriatric Medicine, Graduate School of Medical Sciences Kumamoto University Japan; ^5^ Department of Gastroenterology and Hepatology, Graduate School of Medical Sciences Kumamoto University Japan; ^6^ Department of Dermatology and Plastic Surgery, Graduate School of Medical Sciences Kumamoto University Japan; ^7^ Division of Developmental Genetics, Institute of Resource Development and Analysis Kumamoto University Japan; ^8^ International Research Center for Medical Sciences (IRCMS) Kumamoto University Japan; ^9^ Urologic Oncology Branch, Center for Cancer Research National Cancer Institute Bethesda MD USA

**Keywords:** ANGPTL2, H3K27me3, MHC‐I, PRC2, tumor immune evasion

## Abstract

Loss or downregulation of major histocompatibility complex class I (MHC‐I) contributes to tumor immune evasion. We previously demonstrated that angiopoietin‐like protein 2 (ANGPTL2) promotes tumor progression using a Xp11.2 translocation renal cell carcinoma (tRCC) mouse model. However, molecular mechanisms underlying ANGPTL2 tumor‐promoting activity in the tRCC model remained unclear. Here, we report that ANGPTL2 deficiency in renal tubular epithelial cells slows tumor progression in the tRCC mouse model and promotes activated CD8^+^ T‐cell infiltration of kidney tissues. We also found that *Angptl2*‐deficient tumor cells show enhanced interferon γ‐induced expression of MHC‐I and increased susceptibility to CD8^+^ T‐cell‐mediated anti‐tumor immune responses. Moreover, we provide evidence that the ANGPTL2‐α5β1 integrin pathway accelerates polycomb repressive complex 2‐mediated repression of MHC‐I expression in tumor cells. These findings suggest that ANGPTL2 signaling in tumor cells contributes to tumor immune evasion and that suppressing that signaling in tumor cells could serve as a potential strategy to facilitate tumor elimination by T‐cell‐mediated anti‐tumor immunity.

## Graphical abstract

The ANGPTL2–α5β1‐integrin pathway in tumor cells accelerates polycomb repressive complex 2‐mediated repressive histone modification at the major histocompatibility complex class I (MHC‐I) promoter via JARID2 induction, thereby attenuating interferon γ‐induced MHC‐I expression in tumor cells. As a result, tumor cells show reduced susceptibility to CD8^+^ T cell‐mediated anti‐tumor immune responses, allowing tumor progression. Our findings provide novel insight into mechanisms underlying tumor immune evasion.

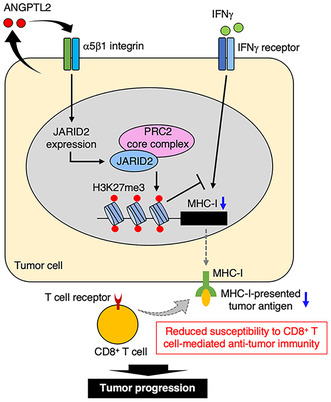

**Abbreviations**
ANGPTL2: angiopoietin‐like protein 2
B2M: β2‐macroglobulin
ccRCC: clear cell renal cell carcinoma
ChIP: chromatin immunoprecipitation
CKO: conditional KO
Eed: embryonic ectoderm development
EpCAM: epithelial cell adhesion molecule
Ezh2: enhancer of zeste homolog 2
FCS: fetal calf serum
H3K27me3: tri‐methylation of histone H3 lysine 27
H3K4me3: tri‐methylation of histone H3 lysine 4
HLA: human leucocyte antigen
HRP: horseradish peroxidase
ICGC: international cancer genome consortium
ICIs: immune checkpoint inhibitors
IFNγ: interferon γ
Jarid2: jumonji and AT‐rich interaction domain containing 2
MHC‐I: major histocompatibility complex class I
Mtf2: metal response element binding transcription factor
NFAT: nuclear factor of activated‐T cells
OVA: ovalbumin
PD‐1: programed cell death‐1
PIR‐B: paired immunoglobulin‐like receptor B
Plat‐E: Platinum‐E
PRC2: polycomb repressive complex 2
STAT1: signal transducer and activator of transcription 1
TAPBP: TAP binding protein
TCR: T cell receptor
TGCA: The Cancer Genome Atlas
TGF‐β: tumor growth factor‐β
TKIs: tyrosine kinase inhibitors
TNFα: tumor necrosis factor α
tRCC: translocation renal cell carcinoma

## 1 Introduction

Immune surveillance is essential for suppression of tumor development and progression. Various types of immune cells function in cancer immune surveillance, and in particular, CD8^+^ cytotoxic T cells play critical roles in T‐cell‐mediated anti‐tumor immunity [1, 2, 3]. CD8^+^ cytotoxic T cells recognize tumor cells displaying tumor antigens bound to major histocompatibility complex class I (MHC‐I)/human leucocyte antigen (HLA) proteins and kill tumor cells by producing cytotoxic effector molecules, such as granzyme B and tumor necrosis factor α (TNFα) [1, 2, 3].

It is well known that expression of MHC‐I and antigen presentation machinery‐related genes, such as *β2‐macroglobulin* (*B2M*), *TAP1/2*, and *TAP‐binding protein* (*TAPBP*), is induced by interferon γ (IFNγ) signaling [4, 5, 6]. IFNγ activates signal transducer and activator of transcription 1 (STAT1) via Janus kinase 1/2, and in turn phosphorylated STAT1 homodimers induce expression of the transcriptional activators IRF1 and NLRC5 [5, 6]. NLRC5 forms an enhanceosome with various transcriptional regulators, which then with IRF1 cooperatively activates transcription of MHC‐I and antigen presentation machinery‐related genes [5, 6].

Mutations of MHC‐I molecules and genes encoding components of the IFNγ signaling pathway are found in various cancers [4, 7, 8]. Tumor cells harboring such mutations exhibit decreased tumor immunogenicity and/or IFNγ insensitivity, leading to evasion of T‐cell‐mediated anti‐tumor immunity and resistance to immunotherapy [4, 7, 8]. Furthermore, changes in either cellular metabolism or epigenetic activities reportedly contribute to downregulation of MHC‐I molecules or reduced tumor cell responsiveness to IFNγ [9, 10]. However, these mechanisms are not well understood.

Xp11.2 translocation renal cell carcinoma (tRCC) is a rare sporadic kidney cancer caused by various *TFE3* gene fusions [11, 12]. TFE3 chimeric proteins are constitutively active transcription factors that induce expression of TFE3 target genes, leading to tRCC development and progression [11, 12]. We previously demonstrated that TFE3 chimeric proteins induce expression of angiopoietin‐like protein 2 (ANGPTL2) [13], a secretory protein that accelerates tumor progression in some cancers, such as lung, breast and colorectal cancers, and osteosarcoma [14, 15, 16, 17]. We also established a tRCC mouse model in which the *PRCC‐TFE3* fusion gene is constitutively expressed in renal tubular epithelial cells [18, 19] and demonstrated that tumor cell‐derived ANGPTL2 is tumor‐promoting, whereas tumor stroma‐derived ANGPTL2 suppresses tumor progression by enhancing dendritic cell activation and subsequent CD8^+^ T‐cell‐mediated anti‐tumor immune responses [13]. However, it remains unclear how tumor cell‐derived ANGPTL2 accelerates tumor progression in the context of tRCC.

Here, we report that tumor cell‐derived ANGPTL2 contributes to tumor progression by accelerating epigenetic repression of MHC‐I expression in tumor cells, resulting in their evasion of CD8^+^ T‐cell‐mediated anti‐tumor immune responses. Our findings provide novel insight into tumor immune evasion.

## 2 Materials and methods

### 2.1 Animals

All experimental procedures were approved by the Kumamoto University Ethics Review Committee for Animal Experimentation (approval no. A2022‐031). Genetically engineered mice used in this study were: *Rosa* ^*LSL‐PRCC‐TFE3/+*^ mice [19] on a C57BL/6N background, Tg mice overexpressing *Cre* driven by the murine *Cadherin 16* promoter (*Cdh16*‐*Cre*) [20] on a C57BL/6N background, *Angptl2* ^*Flox/Flox*^ mice [21] on a C57BL/6N background, and OT‐I T cell receptor (TCR) Tg mice [22] on a C57BL/6J background. *Rosa* ^*LSL‐PRCC‐TFE3/+*^ mice and *Angptl2* ^*Flox/Flox*^ mice were generated as described previously [19, 21]. *Cdh16*‐*Cre* Tg mice were kindly provided by P. Igarashi (The University of Texas Southwestern Medical Center, USA). OT‐I TCR Tg mice were obtained from The Jackson Laboratory (Bar Harbor, ME, USA).

To generate *Cdh16*‐*Cre*; *Rosa26* ^*LSL‐PRCC‐TFE3*/+^ (tRCC) mice, *Cdh16*‐*Cre* mice were mated with *Rosa26* ^*LSL‐PRCC‐TFE3/+*^ mice. To generate *Cdh16*‐*Cre*; *Rosa* ^*LSL‐PRCC‐TFE3/+*^; *Angptl2* ^*Flox/Flox*^ (conditional KO (CKO) tRCC) mice, *Cdh16*‐*Cre*; *Angptl2* ^*Flox/Flox*^ mice were mated with *Rosa* ^*LSL‐PRCC‐TFE3/+*^; *Angptl2* ^*Flox/Flox*^ mice.

All animals were fed a normal diet (CE‐2; CLEA, Tokyo, Japan), bred in a mouse house with automatically controlled lighting (12 h on, 12 h off), and maintained at a stable temperature of 22 ± 2 °C and relative humidity 40–80%.

### 2.2 Cell culture

The mouse primary tRCC line R286, which was established from tRCC mouse tumors [19], and the human tRCC line UOK120 (RRID:CVCL_B099), which was established from tumor tissue of a patient with tRCC [23], were maintained in DMEM supplemented with 10% fetal calf serum (FCS), 100 U·mL^−1^ penicillin, and 100 μg·mL^−1^ streptomycin under 5% CO_2_ and 95% air. The retrovirus packaging cell line Platinum‐E (Plat‐E) (RRID:CVCL_B488) (Cell Biolabs Inc., San Diego, CA, USA) was maintained in DMEM supplemented with 10% FCS, 1 μg·mL^−1^ puromycin, and 10 μg·mL^−1^ blasticidin. The human renal tubular epithelial cell line HK‐2 (RRID:CVCL_0302) (ATCC, Manassas, VA, USA) harboring *HA*‐*PRCC‐TFE3* (HK‐2/HA‐PRCC‐TFE3) [13] was maintained in DMEM/F‐12 supplemented with 10% FCS, 0.8 μg·mL^−1^ puromycin, and 10 μg·mL^−1^ blasticidin. All cell lines have been authenticated in the past 3 years by short tandem repeat analysis using the PowerPlex 16 HS System (Promega, Madison, WI, USA). All experiments were performed with mycoplasma‐free cells.

### 2.3 Establishment of knockout cell lines

Knockout lines were established using a Guide‐it CRISPAR/Cas9 system (Takara Bio, Shiga, Japan) according to the manufacturer's instructions. In brief, mouse *Angptl2* or *Itgα5*‐targeting sgRNAs were designed using the web tool CHOPCHOP [24]. Sense and antisense oligos that correspond to each sgRNA (Table S1) were annealed and cloned into the pGuide‐it ZsGreen1 vector (Takara Bio). To construct the plasmid harboring control sgRNA, Guide‐it control annealed oligos (Takara Bio) were cloned into the vector. Mouse tRCC cells were transfected with plasmids harboring *Angptl2‐* or *Itgα5*‐targeting sgRNAs or control sgRNA using Lipofectamine 3000 reagent (Thermo Fisher Scientific Inc., Waltham, MA, USA) according to the manufacturer's instructions and cultured for 48 h. Cells were harvested and ZsGreen1‐positive cells were isolated using a cell sorter SH800S (SONY, Tokyo, Japan). Established cell clones were subjected to immunoblot analysis or flow cytometry analysis to confirm knockout of target genes.

### 2.4 Establishment of OVA‐ or JARID2‐overexpressing lines

Chicken *OVA* cDNA or mouse *Jarid2* cDNA was cloned into the pMYs‐IRES‐GFP retrovirus vector (Cell Biolabs Inc.). To produce retrovirus, Plat‐E cells were transfected with retrovirus plasmids encoding either chicken *OVA* or mouse *Jarid2* or the pMYs‐IRES‐GFP vector alone using Lipofectamine 3000 reagent (Thermo Fisher Scientific Inc.) and cultured for 48 h, and then culture supernatants containing retrovirus vectors were collected and used to transduce tRCC or *Angptl2* KO tRCC cells, and resulting GFP‐positive cells were isolated using a SH800S cell sorter (SONY). Established clones were subjected to flow cytometry or immunoblot analysis to confirm OVA or JARID2 overexpression.

### 2.5 Histological analysis, immunofluorescence

Kidney tissue from 45 to 49 weeks old male Control, tRCC, and CKO tRCC mice was fixed in 4% paraformaldehyde, embedded in paraffin, and cut into 4‐μm‐thick sections. Sections were deparaffinized, stained with hematoxylin and eosin, and analyzed using a BZ‐X710 microscope (Keyence, Osaka Japan).

For immunofluorescence, 8‐μm‐thick frozen sections of kidney tissues from 40 to 50 weeks old male Control, tRCC, and CKO tRCC mice were fixed in pre‐chilled acetone for 20 min, washed with Tris‐buffered saline (TBS) (pH 7.6) containing 0.05% Triton X‐100 (TBST), and blocked 30 min with 5% normal goat serum. Sections were incubated overnight at 4 °C with following antibodies: anti‐mouse CD8 (1 : 200, #558733; BD Biosciences, San Jose, CA, USA), anti‐mouse H‐2Kb (1 : 100, #116501; Biolegend, San Diego, CA, USA), anti‐TAP1 (1 : 100, #11114‐1‐AP; Proteintech, Rosemont, IL, USA), and anti‐JARID2 (1 : 100, #NB100‐2214; Novus Biologicals, Centennial, CO, USA). After TBST washing, samples were incubated 1 h with Alexa Fluor 594‐conjugated anti‐rat IgG (1 : 500, #A21209; Thermo Fisher Scientific Inc.), Alexa Fluor 594‐conjugated anti‐mouse IgG (1 : 500, #A21203; Thermo Fisher Scientific Inc.), or Alexa Fluor 594‐conjugated anti‐rabbit IgG (1 : 500, #A21207; Thermo Fisher Scientific Inc.) antibodies at room temperature. Nuclei were counterstained with NucBlue Fixed Cell ReadyProbes Reagent (Thermo Fisher Scientific Inc.). Images were obtained using a BZ‐X710 microscope (Keyence).

### 2.6 Kidney tissue digestion for flow cytometry analysis

Kidney tissue from 45 to 52 weeks old male tRCC and CKO tRCC mice was minced and digested with 0.5 mg·mL^−1^ collagenase type 3 (Worthington Biochemical Corporation, Lakewood, NJ, USA), 0.8 ng·mL^−1^ dispase II (Roche, Mannheim, Germany), 0.1 mg·mL^−1^ DNase I (Roche) at 37 °C for 30 min. Tissue was then passed through a 100‐μm cell strainer by mechanical disruption. After centrifugation, the pellet was suspended in red‐blood‐cell lysis buffer (160 mm NH_4_Cl, 10 mm KHCO_3_, and 0.1 mm EDTA) and incubated 5 min at 4 °C. The cell suspension was centrifuged and resuspended in MACS buffer (Miltenyi Biotec, Bergisch Gladbach, Germany).

### 2.7 Flow cytometry analysis

For flow cytometry analysis of *Angptl2* or *Itgα5* KO mouse tRCC lines, cells were incubated 24 h in the presence or absence of 2 ng·mL^−1^ recombinant murine IFNγ (Wako, Osaka, Japan). For some experiments, the OVA‐overexpressing mouse tRCC line was treated 5 days with 3 μm EPZ011989 (Selleck Biotech, Tokyo, Japan), which is an enhancer of zeste homolog 2 (EZH2) inhibitor, and then incubated 24 h with 2 ng·mL^−1^ recombinant murine IFNγ (Wako) in the presence of 3 μm EPZ011989. Cells were then treated with accutase (Sigma‐Aldrich, St Louis, MO, USA), collected, and suspended in MACS buffer (Miltenyi Biotec).

Mouse cells were incubated with Ultra‐LEAF Purified anti‐mouse CD16/32 antibody (#101329; Biolegend) for Fc receptor blocking. Cells were stained with the following antibodies: FITC anti‐mouse CD3e (#553062; BD Biosciences), PE anti‐mouse CD4 (#553049; BD Biosciences), PerCP/Cy5.5 anti‐mouse CD45 (#103131; Biolegend), APC anti‐mouse CD8a (#100711; Biolegend), PE anti‐human Granzyme B (#561142; BD Biosciences), APC anti‐mouse H‐2Kb (#116518; Biolegend), PE anti‐mouse H‐2Db (#111507; Biolegend), PE anti‐mouse CD49e (#103805; Biolegend), PE anti‐mouse CD29 (#102207; Biolegend), PE anti‐mouse CD49e (#103905; Biolegend), PE anti‐mouse PIR‐B (#FAB2754P; R&D Systems, Minneapolis, MN, USA), PE/Cyanine7 anti‐mouse H‐2Kb bound to SIINFEKL (#141607; Biolegend), Brilliant Violet 421 anti‐mouse CD31 (#102423; Biolegend), and APC anti‐mouse CD326 (EpCAM) (#118213; Biolegend).

For flow cytometry analysis of UOK120 cells, cells were incubated 24 h in the presence or absence of 1 ng·mL^−1^ of recombinant human IFNγ (Wako). Cells were then treated with accutase (Sigma‐Aldrich), collected, and suspended in MACS buffer (Miltenyi Biotec). Cells were incubated with 5 μg·mL^−1^ normal goat IgG for Fc blocking and stained with PE anti‐human HLA‐A/B/C (#311405; Biolegend).

Stained cells were analyzed by BD FACSVerse (BD Biosciences). Data analysis was performed using flowjo software (BD Biosciences).

### 2.8 *In vitro* activation and isolation of CD8^+^ OT‐I T cells

Splenocytes harvested from the spleen of 12 weeks‐old male OT‐I TCR Tg mice were incubated 24 h in medium (RPMI‐1640 supplemented with 10% FCS, 1 mm pyruvate, 50 μm 2‐mercaptoethanol, 100 U·mL^−1^ penicillin, and 100 μg·mL^−1^ streptomycin) containing 300 ng·mL^−1^ OVA (SIINFEKL) peptide (MBL, Tokyo, Japan) to activate CD8^+^ OT‐I T cells. Cells were then maintained in medium containing 100 U·mL^−1^ recombinant murine IL‐2 (PeproTech, Cranbury, NJ, USA) for 48 h. Medium was changed and then cells were cultured in medium containing 100 U·mL^−1^ murine IL‐2 for an additional 74 h. Activated CD8^+^ OT‐I T cells were isolated using a CD8a^+^ T‐cell isolation kit (Miltenyi Biotec) according to the manufacturer's instructions.

### 2.9 T‐cell cytotoxicity assay

Ovalbumin‐overexpressing or control tumor cells were seeded at 1 × 10^4^ cells/well in 96‐well plates. OT‐I cells were added at different effector : target ratios (E : T ratios) and incubated 8 h at 37 °C. Cytotoxicity was then assessed using a CytoTox 96 Non‐Radioactive Cytotoxicity Assay (Promega) according to the manufacturer's instructions. For some experiments, OVA‐overexpressing tumor cells were treated 6 days with 3 μm EPZ011989 (Selleck Biotech) prior to the assay.

### 2.10 Measurement of T‐cell cytokines

1 × 10^4^ control or OVA‐overexpressing tumor cells were co‐cultured 8 h with 8 × 10^4^ OT‐I cells in 96 well plates at 37 °C. Culture supernatants were then collected and IFNγ and TNFα levels were measured using an ELISA MAX Deluxe Set (Biolegend) according to the manufacturer's instructions.

### 2.11 Quantitative real‐time PCR analysis

Mouse tRCC cells were incubated 4 h in the presence or absence of 2 ng·mL^−1^ recombinant murine IFNγ (Wako). For some experiments, mouse tRCC cells were treated 6 days with 3 μm EPZ011989 (Selleck Biotech) and then incubated 4 h in the presence or absence of 2 ng·mL^−1^ recombinant murine IFNγ (Wako). After IFNγ stimulation, cells treated with TRI Reagent (Molecular Research Center, Cincinnati, OH, USA) for RNA isolation.

To induce fusion gene expression, HK‐2/HA‐PRCC‐TFE3 cells were cultured 48 h in presence of 100 ng·mL^−1^ doxycycline and treated with TRI Reagent (Molecular Research Center).

Total RNA was isolated using an RNeasy Mini Kit (Qiagen, Valencia, CA, USA). Reverse transcription reactions and PCR were performed using PrimeScript RT Master Mix (TaKaRa Bio) and TB Green Premix Ex Taq II (TaKaRa Bio), respectively. Primer pairs are shown in Table S2. PCR products were analyzed using a Thermal Cycler Dice Real Time System (TaKaRa Bio). Relative transcript abundance was normalized to that of *18S* rRNA levels.

### 2.12 Immunoblot analysis

*Angptl2* or *Itgα5* KO mouse tRCC lines were incubated 4 h with 0.5, 2, or 8 ng·mL^−1^ of recombinant murine IFNγ (Wako). Cells were then collected and homogenized in RIPA buffer (50 mm Tris–HCl, 150 mm NaCl, 0.5% sodium deoxycholate, 1% SDS, 1% NP‐40, 1 mm EDTA, 10 mm NaF, 2 mm Na_3_VO_4_, 10 mm Na_4_P_2_O_7_, plus cOmplete, EDTA‐free protease inhibitor cocktail (Roche), pH 7.5). Samples were centrifuged, and supernatants served as protein extracts, which were subjected to SDS‐polyacrylamide gel electrophoresis. Proteins were electrotransferred to PVDF membrane, and immunoblotted with antibodies against ANGPTL2 (BAF1444; R&D Systems), phosphorylated STAT1 (Y701) (#7649; Cell Signaling Technology, Danvrse, MA, USA), STAT1 (#14994; Cell Signaling Technology), IRF1 (#8478; Cell Signaling Technology), EZH2 (#5246; Cell Signaling Technology), JARID2 (#13594; Cell Signaling Technology), HA‐tag (#3724; Cell Signaling Technology), and Hsc70 (sc‐7298; Santa Cruz Biotechnologies, Santa Cruz, CA, USA). Antibodies were diluted 1 : 2000, and membranes were incubated at 4 °C overnight. After PBST washing, membranes were incubated 1 h with 1 : 3000 diluted horseradish peroxidase (HRP)‐conjugated anti‐rabbit IgG (#7074; Cell Signaling Technology) or HRP‐conjugated sheep anti‐mouse IgG (NA9310; GE Healthcare Life Science, Piscataway, NJ, USA) antibodies at room temperature. Immunodetection was carried out using ECL Prime Western Blotting Detection Reagents or ECL Western Blotting Detection Reagents (both from Cytiva, Tokyo, Japan).

### 2.13 ANGPTL2 KD in human tRCC cells

UOK120 cells were transfected with Mission siRNA Universal Negative Control (SIC001; Sigma‐Aldrich) or human *ANGPTL2*‐targeting siRNA (s23854: GAGAGUUCAUUUACCUAAATT; Thermo Fisher Scientific Inc.) using Lipofectamine RNAi MAX reagent (Thermo Fisher Scientific Inc.) according to the manufacturer's instructions. The medium was changed 16 h after transfection, and cells were incubated an additional 48 h. Cells were then harvested and subjected to immunoblot or flow cytometry analyses. In some experiments, siRNA‐transfected cells were incubated 12 h in the presence or absence of 1 ng·mL^−1^ recombinant human IFNγ (Wako) and treated with TRI Reagent (Molecular Research Center) for quantitative real‐time PCR analysis.

### 2.14 Chromatin immunoprecipitation assay

Chromatin immunoprecipitation (ChIP) analysis was performed as described [25]. In brief, doxycycline‐treated or ‐untreated HK‐2/HA‐PRCC‐TFE3 cells were fixed 5 min with 1% formalin at room temperature. In some experiments, *Angptl2* KO or *Itgα5* KO mouse tRCC lines were incubated with 1 ng·mL^−1^ of recombinant murine IFNγ (Wako) for 4 h and fixed with 1% formalin for 5 min at room temperature. Crosslinking reactions were stopped by incubation with 125 mm glycine. Cells were harvested and treated 30 min with micrococcal nuclease (New England Bio Labs, Ipswich, MA, USA) at 37 °C. Cells were then lysed and lysates served as chromatin samples, which were incubated with anti‐HA‐tag antibody (#3724; Cell Signaling Technology), anti‐H3K27me3 antibody (#9733; Cell Signaling Technology), anti‐H3K4me3 antibody (#9751; Cell Signaling Technology), anti‐EZH2 antibody (#5246; Cell Signaling Technology), or control normal rabbit IgG (#2729; Cell Signaling Technology) overnight at 4 °C. Chromatin complexes were then precipitated with Protein G magnetic beads (Cell Signaling Technology). After washing, DNA was purified from samples and subjected to real‐time PCR analysis. Primer pairs are shown in Table S3.

### 2.15 Analysis of gene expression datasets from human cancer lines or cancer patients

A gene expression dataset of human cancer lines (Table S4) was obtained from the public database Cancer Dependency Map (https://depmap.org/portal/). Correlations between *ANGPTL2* and *JARID2*, *HLA‐B*, or *HLA‐C* expression levels in 636 primary lesion‐derived lines and 424 metastatic lesion‐derived lines were analyzed using graphpad prism 7 software (version 7.03; GraphPad software, La Jolla, CA, USA).

Gene expression datasets of the International Cancer Genome Consortium (ICGC)/The Cancer Genome Atlas (TGCA) pan‐cancer whole genome analysis (*n* = 1210 samples, 23 tumor types) [26] (Table S5), TGCA clear cell renal cell carcinoma (ccRCC) (*n* = 417 samples) [27] (Table S6), and TGCA metastatic melanoma (*n* = 121 patients) [28] (Table S7) were obtained from the public database cBioPortal for Cancer Genomics (http://www.cbioportal.org) [29, 30]. Correlations between expression of indicated genes were analyzed using graphpad prism 7 software (GraphPad software).

### 2.16 Statistical analysis

Statistical analyses were performed using graphpad prism 7 software (version 7.03; GraphPad software). Data were represented as means ± SD. Results with *P*‐values < 0.05 were considered significant. Comparisons between two groups were performed using two‐sided unpaired Student's *t*‐tests. Comparisons between three or more groups were performed using one‐way ANOVA followed by Tukey's, Dunnett's, or Sidak's *post hoc* tests. For comparisons with two or more independent variable factors, we used two‐way ANOVA followed by Tukey's, Dunnett's, or Sidak's *post hoc* tests. Survival rate was analyzed by log‐rank test. To analyze gene expression datasets of human cancer lines and cancer patients, we employed Spearman's test to calculate correlation coefficients used to evaluate relationships between expression levels of genes of interest.

## 3 Results

### 3.1 *Angptl2* deficiency in tumor cells enhances infiltration of kidney tissues by activated CD8^+^ T cells

**Fig. 1:**
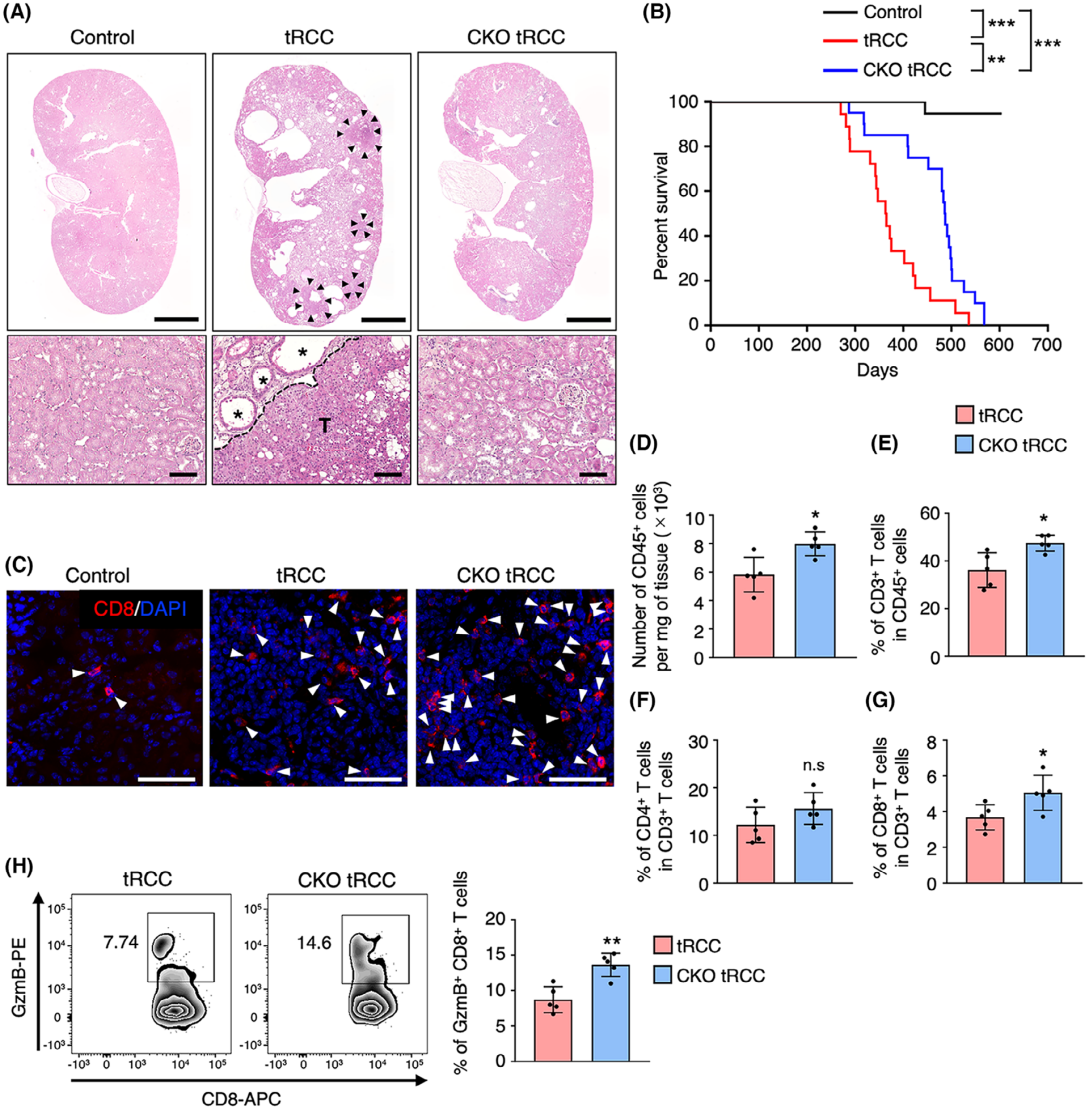
CKO tRCC mice exhibit enhanced infiltration of kidney tissues by activated CD8^+^ T cells. (A) Representative hematoxylin–eosin staining images of kidney tissues from Control (*Rosa* ^*LSL‐PRCC‐TFE3/+*^; *Angptl2* ^*Flox/Flox*^), tRCC (*Cdh16*‐*Cre*; *Rosa26* ^*LSL‐PRCC‐TFE3*/+^), and CKO tRCC (*Cdh16*‐*Cre*; *Rosa* ^*LSL‐PRCC‐TFE3/+*^; *Angptl2* ^*Flox/Flox*^) mice at 45–49 weeks of age. Scale bar, 2 mm (top) and 100 μm (bottom). Shown is a representative of three independent experiments. Arrowheads and asterisks indicate respective tumors and dilated renal tubules. T, tumor lesion. (B) Kaplan–Meier survival curves of control (*n* = 20), tRCC (*n* = 18), and CKO tRCC (*n* = 20) mice. Statistical significance was determined by log‐rank test. **P < 0.01; ***P < 0.001. (C) Immunofluorescent staining for CD8 (red) in kidney tissues from Control, tRCC, and CKO tRCC mice at 40 weeks of age. Shown is a representative of two independent experiments. Nuclei are counterstained with DAPI (blue). Arrowheads indicate CD8^+^ T cells. Scale bar, 100 μm. (D–G) Flow cytometry analysis of infiltrating lymphocytes in kidney tissues from tRCC and CKO tRCC mice at 45–50 weeks of age (*n* = 5 per group). The number of CD45^+^ cells (D) is shown per mg tissue. Frequency of CD3^+^ T (E), CD4^+^ T (F), and CD8^+^ T (G) cells is shown as a percentage of total CD45^+^ T cells (E) or total CD3^+^ T cells (F, G). Data are means ± SD. Statistical significance was determined by two‐sided unpaired Student's *t*‐test. *P < 0.05; n.s, not significant. (H) Flow cytometry analysis of granzyme B (GzmB)‐producing CD8^+^ (GzmB^+^ CD8^+^) T cells in kidney tissues from tRCC and CKO tRCC mice at 45–50 weeks of age (*n* = 5 per group). GzmB^+^ CD8^+^ T‐cell frequency is shown as a percentage of total CD8^+^ T cells. Data are means ± SD. Statistical significance was determined by two‐sided unpaired Student's *t*‐test. **P < 0.01.

We previously demonstrated that PRCC‐TFE3 upregulates *ANGPTL2* in renal tubular epithelial cells and that ANGPTL2 accelerates tRCC progression [13]. Consistently, here we found that the human renal tubular epithelial cell line HK‐2 harboring inducible PRCC‐TFE3 showed increased *ANGPTL2* expression relative to uninduced cells (Fig. S1A,B). Moreover, ChIP analysis indicated that PRCC‐TFE3 proteins bind to E‐boxes in the *ANGPTL2* promoter to activate *ANGPTL2* transcription (Fig. S1C). We also confirmed that tRCC pathologies, such as dilated renal tubules and development of renal carcinoma, seen in *Cdh16*‐*Cre*; *Rosa26* ^*LSL‐PRCC‐TFE3*/+^ (tRCC) mice are suppressed in *Cdh16*‐*Cre*; *Rosa* ^*LSL‐PRCC‐TFE3/+*^; *Angptl2* ^*Flox/Flox*^ (CKO tRCC) mice in which ANGPTL2 expression is specifically deleted in renal tubular epithelial cells [13] (Fig. 1A). Survival of both tRCC and CKO tRCC mice was significantly shortened relative to that of *Rosa* ^*LSL‐PRCC‐TFE3/+*^; *Angptl2* ^*Flox/Flox*^ (Control) mice, and CKO tRCC mice showed prolonged survival relative to tRCC mice (Fig. 1B). Together with our previous study [13], these results suggest that tumor cell‐derived ANGPTL2 accelerates tumor progression in tRCC pathology.

Next, we asked how tumor cell‐derived ANGPTL2 promotes tRCC progression. Interestingly, we found that CKO tRCC mice showed increased infiltration of kidney tissues by CD8^+^ T cells relative to tRCC mice (Fig. 1C). We then used flow cytometry to examine lymphocytes infiltrating kidney tissues from tRCC and CKO tRCC mice and observed that the number of CD45^+^ cells infiltrating kidney tissues significantly increased in CKO tRCC compared with tRCC mice (Fig. 1D). Furthermore, CKO tRCC mice showed a significant increase in the CD8^+^ T‐cell population among infiltrating lymphocytes relative to tRCC mice (Fig. 1E–G), suggesting that *Angptl2* deficiency in tumor cells enhances CD8^+^ T‐cell infiltration of kidney tissues. On the other hand, we observed an increase in the CD4^+^ T‐cell population in kidney tissues of CKO tRCC relative to tRCC mice, although those differences were not significant (Fig. 1F). The frequency of cytotoxic effector granzyme B‐expressing CD8^+^ T cells also significantly increased in CKO tRCC relative to tRCC mice (Fig. 1H), suggesting that CD8^+^ T‐cell activation is enhanced in CKO tRCC mice. Taken together, these results suggest that enhanced CD8^+^ T‐cell‐mediated anti‐tumor immune responses slow tumor progression in CKO tRCC mice.

### 3.2 *Angptl2* deficiency accelerates CD8^+^ T‐cell activation by increasing MHC‐I expression in tumor cells

**Fig. 2:**
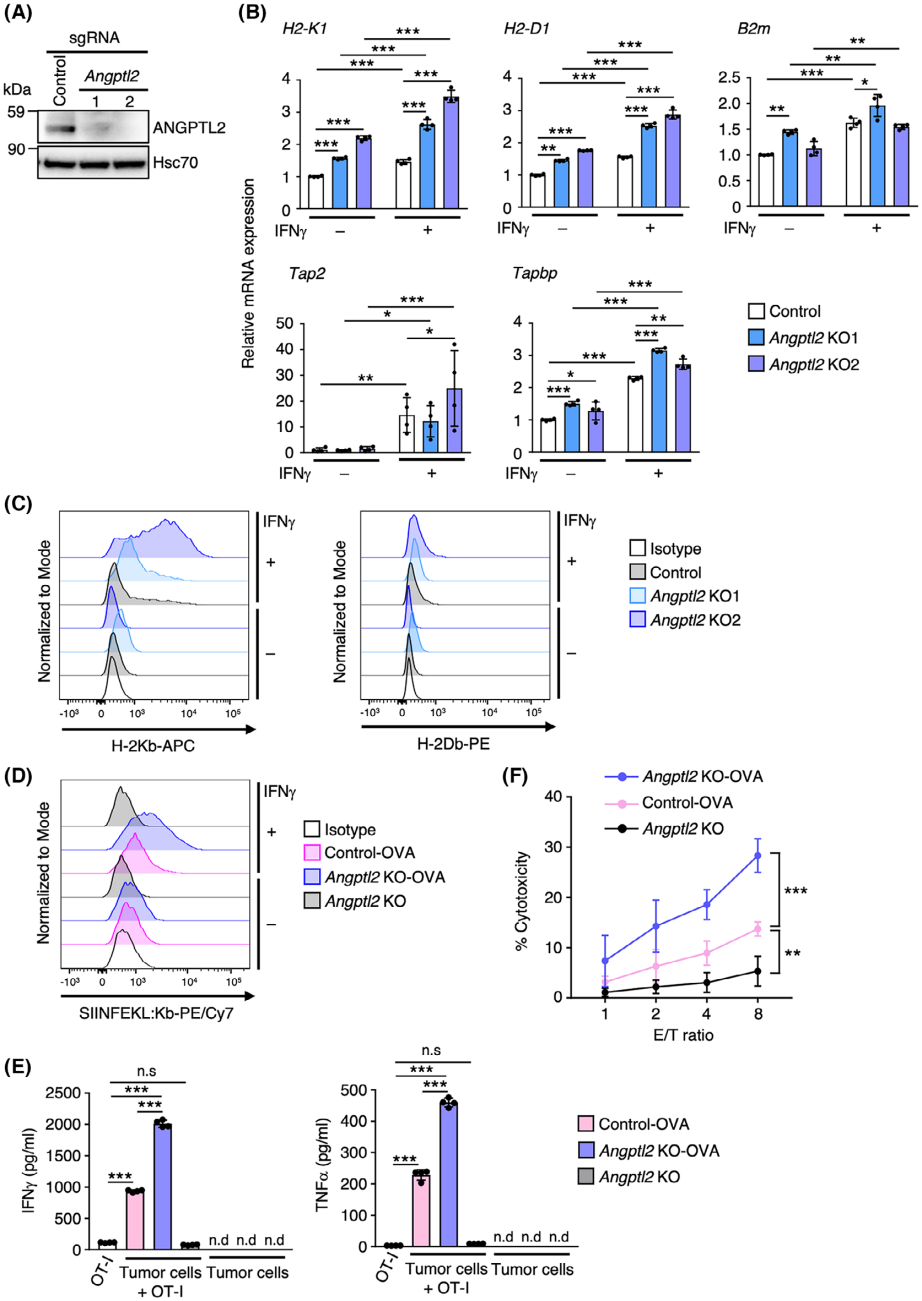
*Angptl2* deficiency accelerates T‐cell‐mediated tumor cell eradication by increasing IFNγ‐induced MHC‐I expression in tumor cells. (A) Representative immunoblotting of ANGPTL2 in control KO (Control) and *Angptl2* KO tRCC cell lines. Shown is a representative of two independent experiments. Hsc70 served as a loading control. (B) Relative expression of mRNAs encoding MHC‐I (*H2‐K1* and *H2‐D1*) and antigen presentation machinery‐related molecules (*B2m*, *Tap2*, and *Tapbp*) in IFNγ‐treated or untreated control KO (Control) and *Angptl2* KO (*Angptl2* KO1 and KO2) cells (*n* = 4 per group). Levels in IFNγ‐untreated control cells were set to 1. Data are means ± SD. Statistical significance was determined by two‐way ANOVA with Tukey's *post hoc* test. *P < 0.05; **P < 0.01; ***P < 0.001. (C) Representative histograms showing cell surface expression of H‐2Kb and H‐2Db in IFNγ‐treated or untreated control KO (Control) and *Angptl2* KO (*Angptl2* KO1 and KO2) cells. Shown is a representative of three independent experiments. (D) Representative histograms showing cell surface expression of SIINFEKL‐bound H‐2Kb in IFNγ‐treated or untreated Control‐OVA, *Angptl2* KO‐OVA, and *Angptl2* KO cells. Shown is a representative of three independent experiments. (E) Levels of indicated secreted T‐cell cytokines (IFNγ and TNFα) in culture supernatants of OT‐I T cells, OT‐I T cells co‐cultured with Control‐OVA, *Angptl2* KO‐OVA, or *Angptl2* KO cells, and in corresponding tumor lines (*n* = 4 per group). Data are means ± SD. Statistical significance was determined by one‐way ANOVA with Tukey's *post hoc* test. ***P < 0.001; n.s, not significant; n.d, not detected. (F) T‐cell cytotoxicity assay with Control‐OVA, *Angptl2* KO‐OVA, and *Angptl2* KO cells (*n* = 6 per group). OT‐I T cells were co‐cultured 8 h with tumor cells at the indicated effector/target (E/T) ratios. Data are means ± SD. Statistical significance was determined by two‐way ANOVA with Dunnett's *post hoc* test. **P < 0.01; ***P < 0.001.

Given that downregulation of the antigen presentation pathway and a reduction in IFNγ sensitivity in tumor cells facilitate evasion of anti‐tumor immunity [8, 9, 10], we hypothesized that tumor cell ANGPTL2 expression regulates these pathways in a way that attenuates CD8^+^ T‐cell‐mediated anti‐tumor immune responses. To investigate this possibility, we established *Angptl2* KO tumor lines (*Angptl2* KO1 and KO2) using the primary tRCC cell line R286 established from tumors of tRCC mice [19] (Fig. 2A) and assessed expression of antigen presentation‐related genes, such as MHC‐I (*H2‐K1* and *H2‐D1*) and antigen presentation machinery‐related molecules (*B2m*, *Tap2*, and *Tapbp*) (Fig. 2B). *Angptl2* KO cells exhibited enhanced expression of antigen presentation‐related genes, especially MHC‐I molecules, compared with control cells in the presence or absence of IFNγ. Flow cytometry analysis also revealed a marked increase in cell surface MHC‐I expression in IFNγ‐stimulated *Angptl2* KO cells (Fig. 2C). These results suggest that *Angptl2* deficiency in tumor cells enhances IFNγ‐induced expression of antigen presentation‐related genes.

Next, we asked whether *Angptl2* deficiency in tumor cells promotes intracellular MHC‐I antigen processing. To do so, we established full‐length chicken Ovalbumin (OVA) protein‐expressing control (control‐OVA) and *Angptl2* KO (*Angptl2* KO‐OVA) tRCC lines (Fig. 2D). Intracellular processing of OVA proteins generates the SIINFEKL peptide, a MHC‐I‐restricted peptide loaded onto MHC‐I (H‐2Kb). IFNγ stimulation induced cell surface expression of SIINFEKL‐bound H‐2Kb (SIINFEKL:Kb) only in OVA‐expressing cells, and that expression in *Angptl2* KO‐OVA cells was significantly higher than that seen in control‐OVA cells (Fig. 2D), suggesting that IFNγ‐induced intracellular MHC‐I antigen processing is accelerated in *Angptl2*‐deficient tRCC cells. To further examine functional consequences of enhanced MHC‐I expression and MHC‐I antigen processing in *Angptl2*‐deficient tRCC cells, we co‐cultured CD8^+^ OT‐I T cells, which express a T‐cell receptor specifically recognizing H‐2Kb‐bound SIINFEKL peptide, with control‐OVA, *Angptl2* KO‐OVA, or *Angptl2* KO cells (Fig. 2E). *Angptl2* KO cells failed to activate co‐cultured OT‐I cells and induce production of T‐cell cytokines (IFNγ and TNFα). On the other hand, T‐cell cytokine production was induced in both control‐OVA and *Angptl2* KO‐OVA cells, and levels of these secreted cytokines were significantly increased in *Angptl2* KO‐OVA relative to control‐OVA cells (Fig. 2E). T‐cell‐mediated killing was also markedly accelerated in *Angptl2* KO‐OVA relative to control‐OVA cells (Fig. 2F). In contrast, *Angptl2* KO cells were resistant to T‐cell‐mediated killing (Fig. 2F). Collectively, these results suggest that ANGPTL2 suppresses IFNγ‐induced MHC‐I expression and intracellular MHC‐I antigen processing to attenuate antigen‐specific T‐cell activation and killing of tumor cells.

### 3.3 ANGPTL2 represses MHC‐I expression in tumor cells via α5β1 integrin

**Fig. 3:**
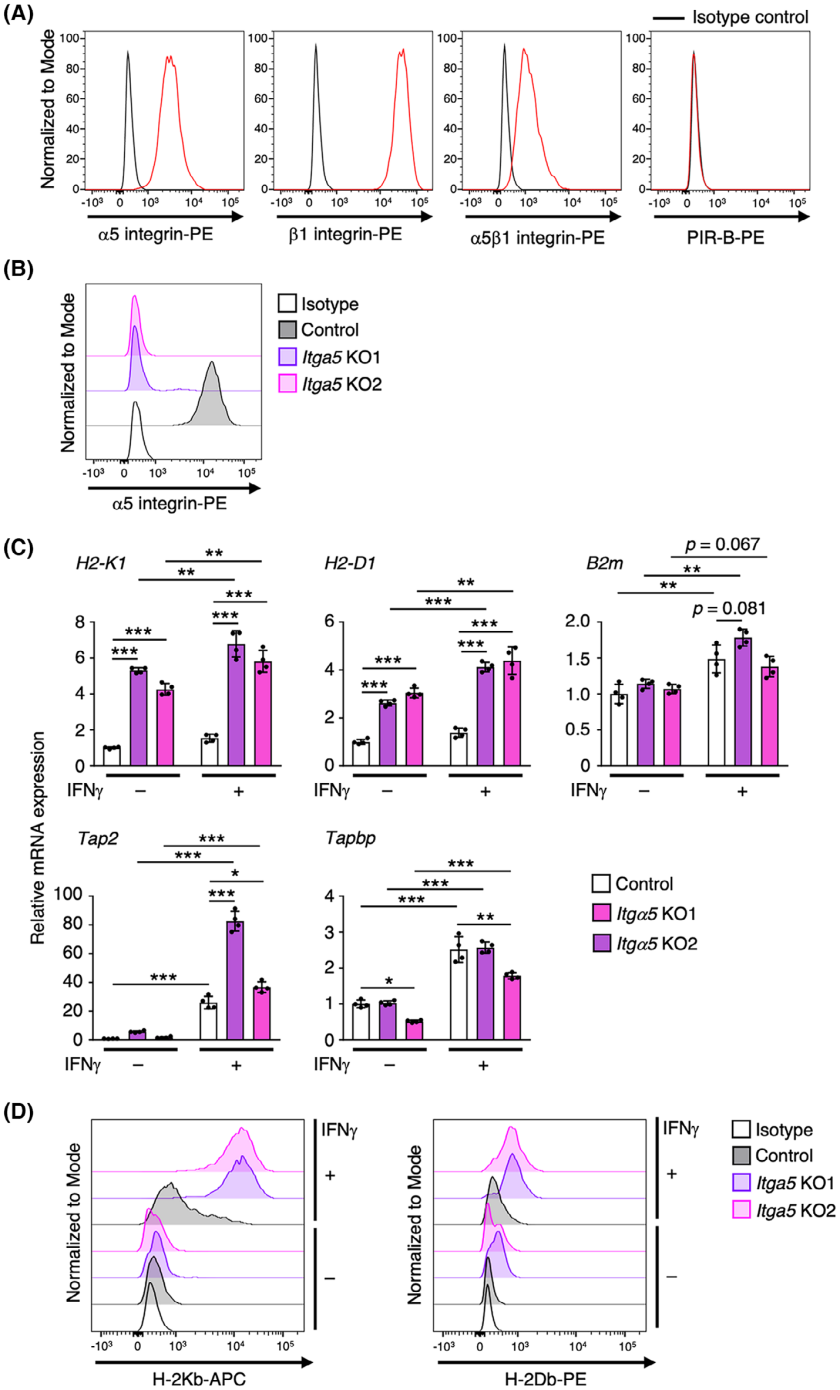
ANGPTL2 attenuates IFNγ‐induced MHC‐I expression via α5β1 integrin. (A) Representative histograms showing cell surface expression of α5 integrin, β1 integrin, α5β1 integrin, and PIR‐B in mouse tRCC cells. Shown is a representative of three independent experiments. (B) Representative histograms showing cell surface expression of α5 integrin in control KO (Control) and *Itgα5* KO (*Itgα5* KO1 and KO2) cells. Shown is a representative of three independent experiments. (C) Relative expression of transcripts encoding MHC‐I (*H2‐K1* and *H2‐D1*) and antigen presentation machinery‐related molecules (*B2m*, *Tap2*, and *Tapbp*) in IFNγ‐treated or untreated control KO (Control) and *Itgα5* KO (*Itgα5* KO1 and KO2) cells (*n* = 4 per group). Levels in IFNγ‐untreated control cells were set to 1. Data are means ± SD. Statistical significance was determined by two‐way ANOVA with Tukey's *post hoc* test. *P < 0.05; **P < 0.01; ***P < 0.001. (D) Representative histograms showing cell surface expression of H‐2Kb and H‐2Db in control KO (Control) and *Itgα5* KO (*Itgα5* KO1 and KO2) cells. Shown is a representative of three independent experiments.

We previously demonstrated that ANGPTL2 promotes tumor progression in some cancers, such as breast cancer, lung cancer, and osteosarcoma, via α5β1 integrin [31]. The paired immunoglobulin‐like receptor B (PIR‐B) also reportedly mediates ANGPTL2 signaling in both leukemic stem and hematopoietic stem cells [32, 33]. Flow cytometry analysis revealed that α5β1 integrin, but not PIR‐B, was expressed in tRCC cells (Fig. 3A). To test whether α5β1 integrin mediates ANGPTL2‐dependent repression of MHC‐I expression in tRCC cells, we established *Itgα5* KO tRCC lines (Fig. 3B). Consistent with results seen in *Angptl2* KO tRCC cells (Fig. 2B), expression levels of MHC‐I and antigen presentation machinery‐related genes were significantly increased in *Itgα5* KO cells relative to control cells, in the presence or absence of IFNγ (Fig. 3C). Moreover, we confirmed a marked increase in IFNγ‐induced cell surface MHC‐I expression in *Itgα5* KO relative to control cells (Fig. 3D). These results suggest that the ANGPTL2‐α5β1 integrin pathway contributes to repression of MHC‐I expression in tumor cells.

### 3.4 ANGPTL2 increases a repressive histone modification at the MHC‐I promoter

**Fig. 4:**
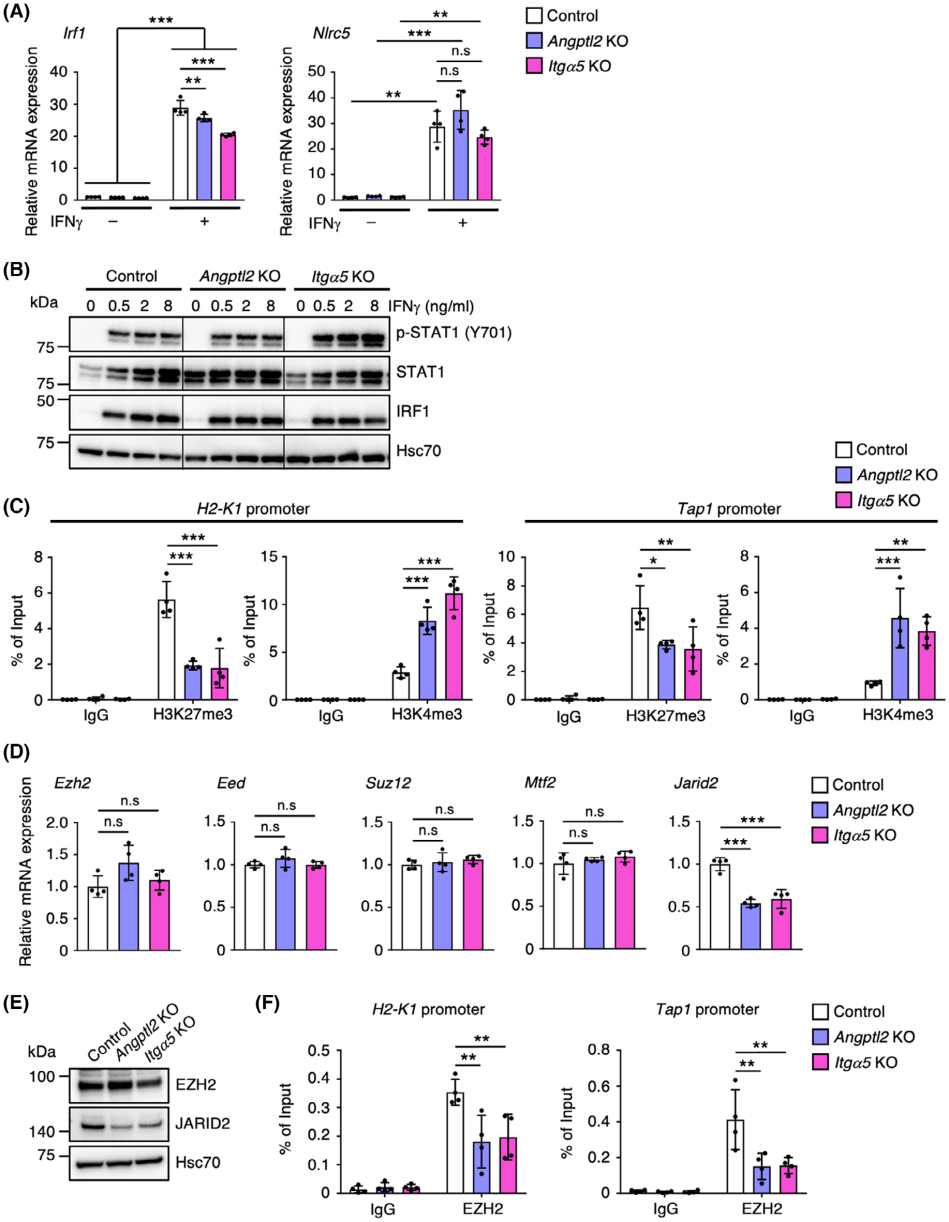
ANGPTL2 promotes H3K27me3‐mediated repression of MHC‐I expression in tumor cells. (A) Relative expression of *Irf1* and *Nlrac5* mRNAs in IFNγ‐treated or untreated control KO (Control), *Angptl2* KO, and *Itgα5* KO cells (*n* = 4 per group). Levels in IFNγ‐untreated control cells were set to 1. Data are means ± SD. Statistical significance was determined by two‐way ANOVA with Tukey's *post hoc* test. **P < 0.01; ***P < 0.001; n.s, not significant. (B) Representative immunoblotting of phosphorylated STAT1 (Y701), total STAT1, and total IRF1 in control KO (Control), *Angptl2* KO, and *Itgα5* KO cells treated with indicated concentrations of IFNγ. Shown is a representative of three independent experiments. Hsc70 served as a loading control. (C) ChIP assay of H3K27me3 and H3K4me3 at the *H2‐K1* and *Tap1* promoters in IFNγ‐treated control KO (Control), *Angptl2* KO, and *Itgα5* KO cells (*n* = 4 per group). Data are means ± SD. H3K27me3 and H3K4me3 levels at each promoter are presented as a percentage of input. Statistical significance was determined by two‐way ANOVA with Dunnett's *post hoc* test. *P < 0.05; **P < 0.01; ***P < 0.001. (D) Relative expression of *Ezh2*, *Eed*, *Suz12*, *Mtf2*, and *Jarid2* mRNAs in control KO (Control), *Angptl2* KO, and *Itgα5* KO cells (*n* = 4 per group). Levels in control cells were set to 1. Data are means ± SD. Statistical significance was determined by one‐way ANOVA with Dunnett's *post hoc* test. ***P < 0.001; n.s, not significant. (E) Representative immunoblotting of EZH2 and JARID2 in control KO (Control), *Angptl2* KO, and *Itgα5* KO cells. Hsc70 served as a loading control. Shown is a representative of three independent experiments. (F) ChIP assay of EZH2 at the *H2‐K1* and *Tap1* promoters in IFNγ‐treated control KO (Control), *Angptl2* KO, and *Itgα5* KO cells (*n* = 4 per group). Data are means ± SD. EZH2 occupancy of each promoter is presented as a percentage of input. Statistical significance was determined by two‐way ANOVA with Dunnett's *post hoc* test. **P < 0.01.

Since IFNγ induces expression of IRF1 and NLRC5, which are key transcriptional activators of various IFNγ‐responsive genes, including MHC‐I and antigen presentation machinery‐related genes, by activating STAT1 [4, 5, 6], we hypothesized that ANGPTL2 attenuates IFNγ‐induced expression of *Irf1* and *Nlrc5* in tRCC cells. To examine this possibility, we analyzed *Irf1* and *Nlrc5* transcript levels in *Angptl2* KO and *Itgα5* KO tRCC cells (Fig. 4A). IFNγ stimulation significantly increased transcript levels of both in control and KO lines. However, although expression levels of MHC‐I and antigen presentation machinery‐related genes significantly increased in KO relative to control cells (Figs 2B and 3C), KO lines did not show increased *Irf1* and *Nlrc5* transcript levels relative to control cells in the presence of IFNγ (Fig. 4A). Furthermore, STAT1 protein phosphorylation was comparably induced in IFNγ‐treated control and KO lines, and IRF1 protein levels were comparable in these lines (Fig. 4B). These results suggest that ANGPTL2 signaling does not alter activation of the IFNγ pathway in tumor cells.

A recent study demonstrated that polycomb repressive complex 2 (PRC2)‐mediated tri‐methylation of histone H3 lysine 27 (H3K27me3) at promoters of MHC‐I and antigen presentation machinery‐related genes silences expression of these genes and contributes to evasion of anti‐tumor immunity by cancers such as lung cancer and neuroblastoma [9]. To assess relevance of these mechanisms to ANGPTL2‐mediated MHC‐I repression in tRCC cells, we assessed levels of H3K27me3 and tri‐methylation of histone H3 lysine 4 (H3K4me3), the latter an active gene expression mark, at the *H2‐K1* and *Tap1* promoters in IFNγ‐treated control, *Angptl2* KO, and *Itgα5* KO tRCC cells using ChIP (Fig. 4C). H3K27me3 levels at both promoters in KO lines were significantly decreased compared to control cells. Conversely, H3K4me3 levels at both promoters were significantly increased in KO lines. These results suggest that ANGPTL2 signaling is associated with accelerated H3K27me3 deposition at promoters of antigen presentation‐related genes.

Next, we asked whether the PRC2 complex functions in ANGPTL2‐mediated repression of MHC‐I expression in tRCC cells. To do so, we first examined effects of EPZ011989, an inhibitor of EZH2, a core subunit of the PRC2 complex [34], on expression of MHC‐I and antigen presentation machinery‐related genes in tRCC cells. EPZ011989‐treated tRCC cells showed enhanced expression of MHC‐I and antigen presentation machinery‐related genes compared with untreated cells in the presence or absence of IFNγ (Fig. S2A). Moreover, EPZ011989‐treated OVA‐expressing tRCC (tRCC‐OVA) cells showed enhanced cell surface expression of SIINFEKL‐bound H‐2Kb compared with untreated tRCC‐OVA cells (Fig. S2B), as well as significantly accelerated T‐cell‐mediated killing of tRCC‐OVA cells (Fig. S2C). These results strongly suggest that the PRC2 complex contributes to repression of MHC‐I expression and decreases T‐cell‐mediated killing in tRCC cells.

We then assessed transcript levels of several factors comprising the PRC2 complex in control, *Angptl2* KO, and *Itgα5* KO cells (Fig. 4D). In tRCC cells, *Angptl2* or *Itgα5* deficiency did not alter expression levels of *Ezh2*, *embryonic ectoderm development* (*Eed*), and *Suz12*, all of which encode core subunits of PRC2 complex [34]. In addition, expression levels of *metal response element binding transcription factor* (*Mtf2*), a non‐core PRC2 complex subunit functioning in complex recruitment to target genes [34, 35], were comparable in those cells. By contrast, relative to control cells, *Angptl2* KO and *Itgα5* KO cells showed significantly decreased expression of the non‐core subunit *jumonji and AT‐rich interaction domain containing 2* (*Jarid2*), which not only recruits the PRC2 complex to target genes but enhances EZH2 methyltransferase activity [34, 35, 36] (Fig. 4D,E). Consistent with our observations of *Angptl2* KO and *Itgα5* KO tRCC lines, CKO tRCC mice also exhibited increased expression of H‐2Kb and TAP1 proteins in tumor lesions from kidney tissues relative to tRCC mice, whereas expression of JARID2 proteins localized to cell nuclei was decreased in CKO tRCC mice (Fig. S3). Furthermore, ChIP analysis revealed significantly reduced EZH2 occupancy of the *H2‐K1* and *Tap1* promoters in KO relative to control lines (Fig. 4F). In addition, JARID2 overexpression in *Angptl2* KO cells significantly suppressed IFNγ‐induced expression of antigen presentation‐related genes, including MHC‐I, and restored EZH2 occupancy of the *H2‐K1* promoter (Fig. S2D–F). Taken together, these results suggest that ANGPTL2 signaling accelerates PRC2 complex recruitment to promoters of antigen presentation‐related genes and subsequent H3K27me3 deposition via JARID2 induction.

### 3.5 The ANGPTL2‐JARID2 axis contributes to repression of HLA class I expression in human cancer cells

**Fig. 5:**
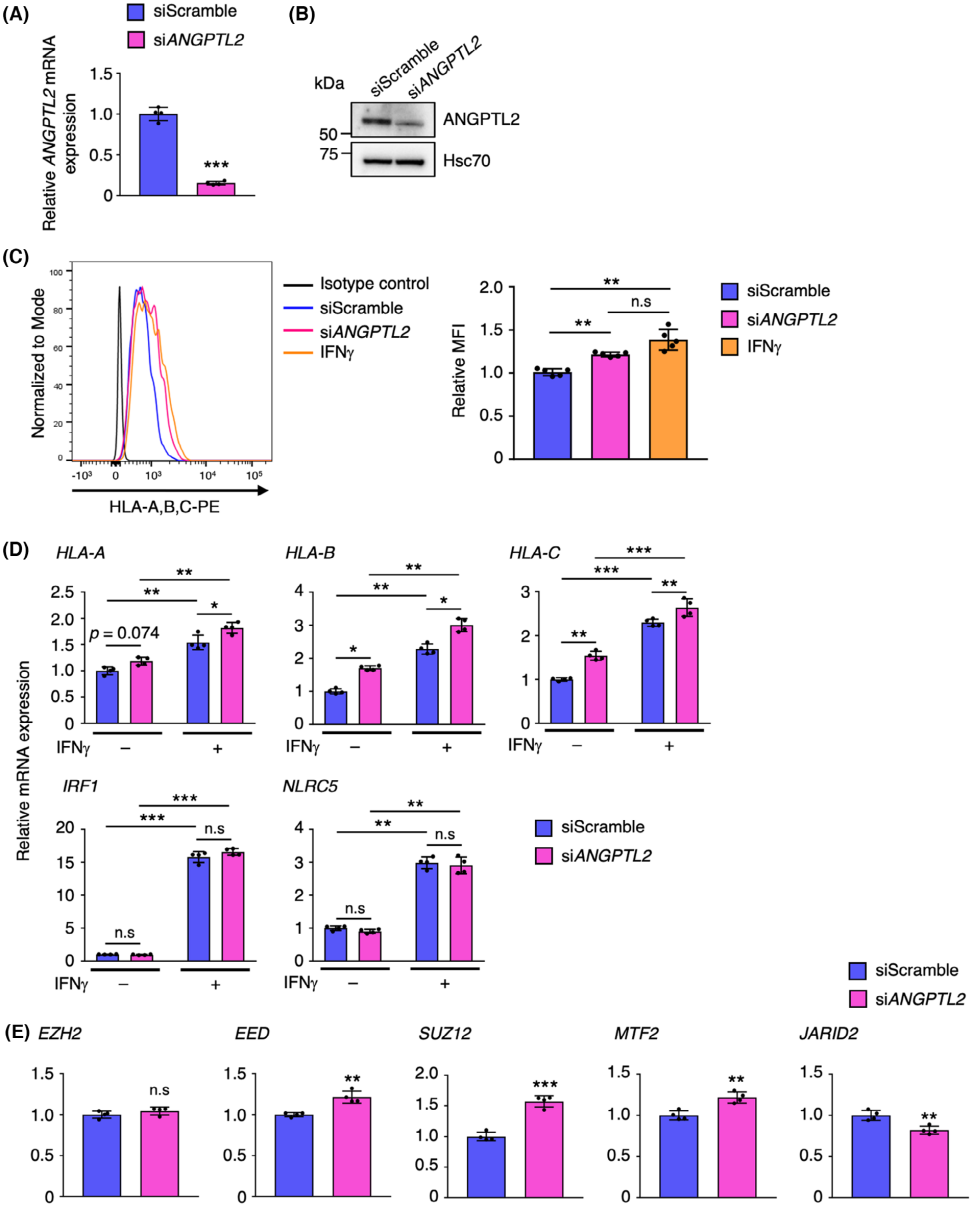
ANGPTL2 knockdown increases MHC‐I expression in human tRCC cells. (A) Relative expression of *ANGPTL2* mRNA in control (siScramble) and *ANGPTL2*‐knockdown (si*ANGPTL2*) UOK120 cells (*n* = 4 per group). Levels in control cells were set to 1. Data are means ± SD. Statistical significance was determined by two‐sided unpaired Student's *t*‐test. ***P < 0.001. (B) Representative immunoblotting of ANGPTL2 in control (siScramble) and *ANGPTL2*‐knockdown (si*ANGPTL2*) UOK120 cells. Shown is a representative of two independent experiments. Hsc70 served as a loading control. (C) Representative histograms showing cell surface expression of HLA class I molecules (HLA‐A, HLA‐B, and HLA‐C) in control (siScramble), *ANGPTL2*‐knockdown (si*ANGPTL2*), and IFNγ‐treated (IFNγ) UOK120 cells (left) and quantification of fluorescent intensity in each group (right) (*n* = 5 per group). Mean fluorescent intensity (MFI) seen in control cells was set to 1. Data are means ± SD. Statistical significance was determined by one‐way ANOVA with Tukey's *post hoc* test. **P < 0.01; n.s, not significant. (D) Relative expression of *HLA‐A*, *HLA‐B*, *HLA‐C*, *IRF1*, and *NLRC5* mRNAs in IFNγ‐treated or untreated control (siScramble) and *ANGPTL2*‐knockdown (si*ANGPTL2*) UOK120 cells (*n* = 4 per group). Levels in IFNγ‐untreated control cells were set to 1. Data are means ± SD. Statistical significance was determined by two‐way ANOVA with Tukey's *post hoc* test. *P < 0.05; **P < 0.01; ***P < 0.001; n.s, not significant. (E) Relative expression of *EZH2*, *EED*, *SUZ12*, *MTF2*, and *JARID2* mRNAs in control (siScramble) and *ANGPTL2*‐knockdown (si*ANGPTL2*) UOK120 cells (*n* = 4 per group). Levels in control cells were set to 1. Data are means ± SD. Statistical significance was determined by two‐sided unpaired Student's *t*‐test. **P < 0.01; ***P < 0.001; n.s, not significant.

To investigate whether ANGPTL2 activity impacts MHC‐I expression in human cancer cells, we performed siRNA‐mediated ANGPTL2 knockdown (KD) in the human tRCC cell line UOK120, which was established from tumors of a tRCC patient [12] (Fig. 5A,B). Flow cytometry analysis revealed that UOK120 cells express HLA class I molecules (HLA‐A, HLA‐B, and HLA‐C) and that their expression levels significantly increased in ANGPTL2 KD relative to control cells (Fig. 5C). Furthermore, expression levels of HLA class I molecules in ANGPTL2 KD cells were comparable to those seen in IFNγ‐treated UOK120 cells (Fig. 5C). ANGPTL2 KD cells also exhibited a significant increase in transcripts encoding HLA class I molecules (*HLA‐A*, *HLA‐B*, and *HLA‐C*) compared with control cells, in the presence or absence of IFNγ (Fig. 5D). By contrast, *IRF1* and *NLRC5* mRNA levels were comparable in ANGPTL2 KD and control cells (Fig. 5D), and there were no differences in *EZH2* expression in ANGPTL2 KD and control cells, while expression levels of *EED*, *SUZ12*, and *MTF2* significantly increased in ANGPTL2 KD relative to control cells (Fig. 5E). In contrast, ANGPTL2 KD cells exhibited significantly decreased expression of *JARID2* compared with control cells (Fig. 5E). Collectively, these results suggest that the ANGPTL2‐JARID2 axis is associated with repression of HLA class I expression in human tRCC cells.

**Fig. 6:**
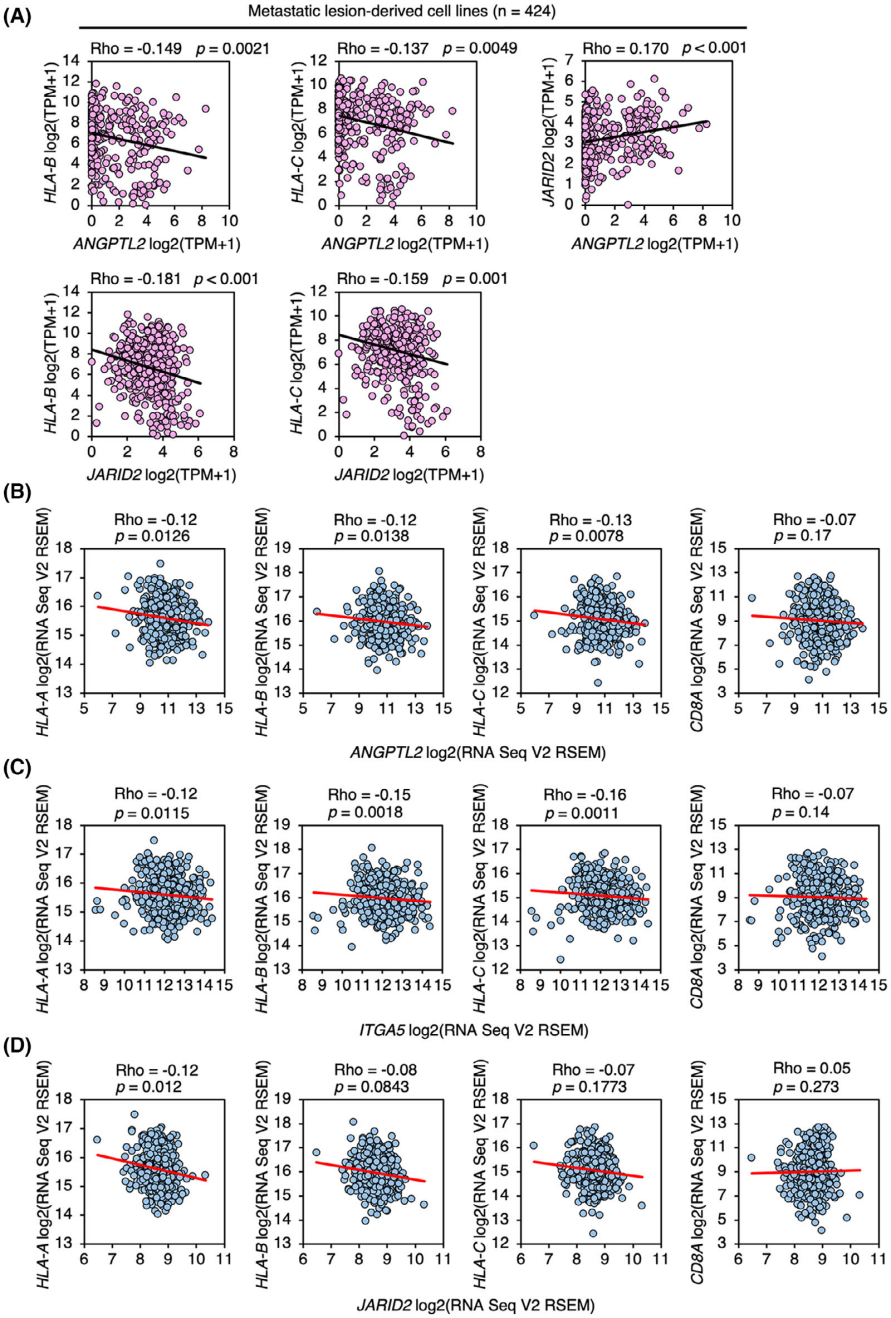
Activation of ANGPTL2 signaling is negatively correlated with expression of HLA class I in human cancer cells. (A) Scatter plots showing correlations between *ANGPTL2*, *HLA‐B*, *HLA‐C*, and *JARID2* mRNA expression levels in metastatic lesion‐derived cell lines. Shown are Spearman's correlation coefficients *r* (Rho). (B–D) Scatter plots showing correlations between *ANGPTL2* (B), *ITGA5* (C), or *JARID2* (D) mRNA levels and those of indicated genes in tumor samples from 417 patients with ccRCC. Shown are Spearman's correlation coefficients *r* (Rho).

To further investigate an association between ANGPTL2 expression and repression of HLA class I expression in human cancer cells, we analyzed a gene expression dataset of 1060 human cancer cell lines, including 636 primary lesion‐derived lines and 424 metastatic lesion‐derived lines, obtained from the public database Cancer Dependency Map (Table S4). In metastatic lines, *ANGPTL2* mRNA levels were negatively correlated with those of *HLA‐B* and *HLA‐C* mRNAs, and conversely, *ANGPTL2* mRNA levels were positively correlated with those of *JARID2* (Fig. 6A). Furthermore, *JARID2* mRNA levels were significantly and negatively correlated with *HLA‐B* and *HLA‐C* mRNA levels (Fig. 6A). All correlations seen in metastatic lines were not observed in primary lesion‐derived cell lines (Fig. S4A).

### 3.6 *ANGPTL2* mRNA levels are negatively correlated with *HLA* class I mRNA levels in patients with clear cell renal cell carcinoma

Analysis of a dataset of the International Cancer Genome Consortium (ICGC)/The Cancer Genome Atlas (TGCA) pan‐cancer whole genome analysis (*n* = 1210 samples, 23 tumor types) [26] (Table S5) revealed a negative correlation of *JARID2* mRNA levels with those of *HLA‐A* and *HLA‐C* mRNAs (Fig. S4B–D). By contrast, we did not observe similar negative correlations of *HLA‐A* and *HLA‐C* mRNAs with *ANGPTL2* and *ITGA5* mRNAs (Fig. S4B–D). In addition, expression levels of *ANGPTL2* and *ITGA5* mRNAs were positively correlated with those of *CD8A* mRNA, and there was no correlation of *JARID2* with *CD8A* mRNA levels (Fig. S4B–D). On the other hand, analysis using a gene expression dataset of TGCA ccRCC (*n* = 417 samples) [27] (Table S6) revealed that *ANGPTL2* and *ITGA5* mRNA levels were negatively correlated with those of *HLA‐A*, *HLA‐B*, and *HLA‐C* mRNAs (Fig. 6B,C). Furthermore, *JARID2* mRNA levels were negatively correlated with *HLA‐A* mRNA levels (Fig. 6D). However, neither *ANGPTL2*, *ITGA5*, nor *JARID2* mRNA levels were correlated with *CD8A* mRNA levels (Fig. 6B–D). These results suggest that at least in ccRCC, the ANGPTL2‐JARID2 axis contributes to repression of HLA class I expression.

Given that defects in the antigen presentation machinery and IFNγ signaling contribute to acquired resistance to immune checkpoint inhibitors (ICIs) [8, 37, 38, 39], we used a TGCA dataset [28] (Table S7) to assess an association between *ANGPTL2* mRNA levels and overall survival after treatment with programed cell death‐1 (PD‐1) inhibitors in patients with metastatic melanoma. We divided patients into two groups based on pre‐treatment biopsy *ANGPTL2* mRNA levels: a high group (*n* = 60) defined as showing higher than median *ANGPTL2* expression, and a low group (*n* = 61), which showed lower than the median value. Patients in the low group showed a shortened overall survival period after treatment with PD‐1 inhibitors compared with the high group (Fig. S5A).

## 4 Discussion

**Fig. 7:**
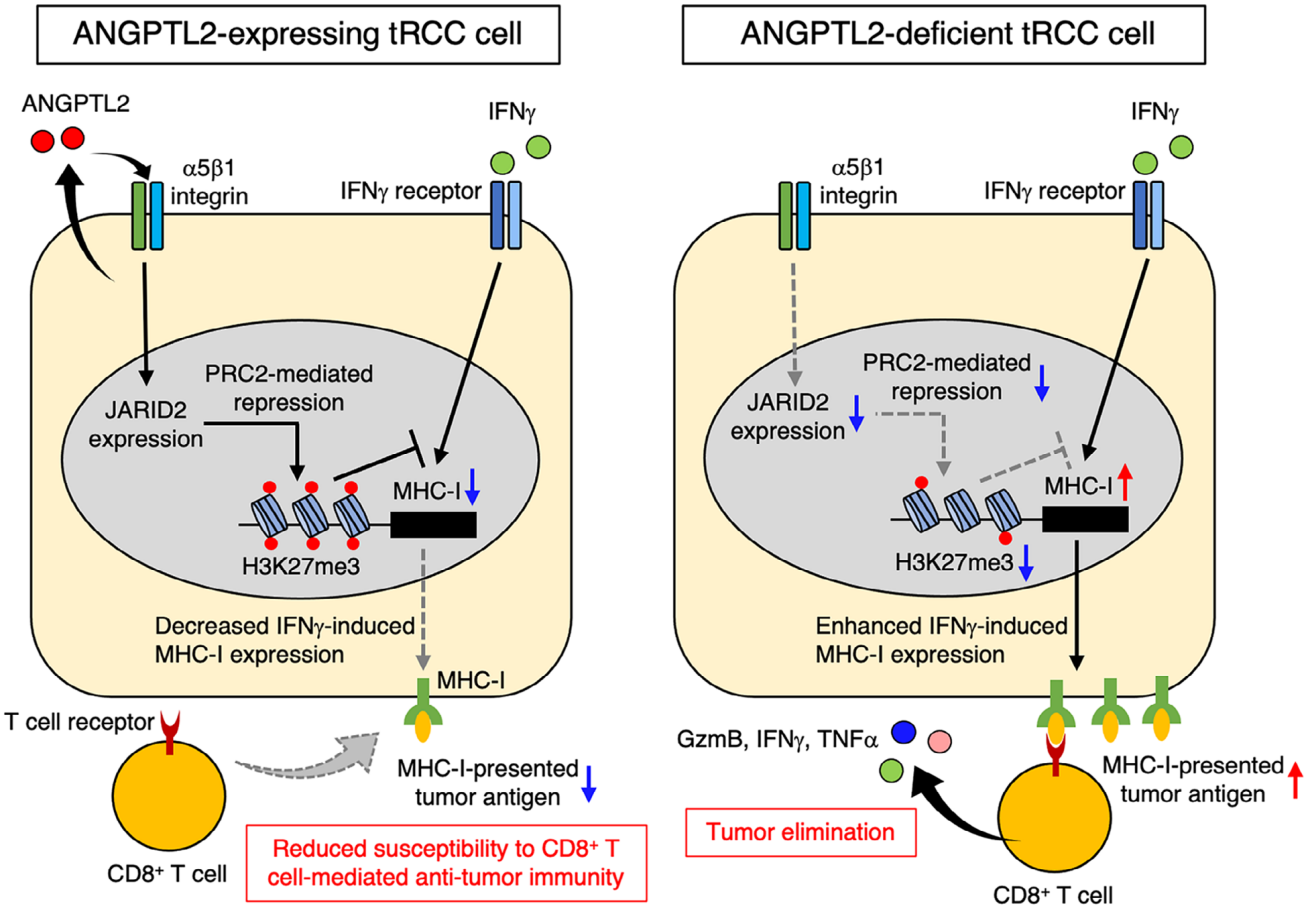
Proposed mechanism underlying ANGPTL2‐mediated evasion of immune eradication by tumor cells. (left) ANGPTL2 activates the α5β1 integrin pathway in tumor cells to induce JARID2 expression and accelerate repressive PRC2‐mediated H3K27me3 deposition at the MHC‐I promoter, thereby repressing IFNγ‐induced MHC‐I expression in tumor cells. As a result, tumor cells show reduced susceptibility to CD8^+^ T‐cell‐mediated anti‐tumor immunity, allowing tumor progression. (right) By contrast, tumor cells lacking ANGPTL2 expression show enhanced responsiveness to IFNγ and upregulation of MHC‐I expression due to decreased JARID2 expression. As a result, ANGPTL2‐deficient tumor cells are more readily eliminated by anti‐tumor CD8^+^ T‐cell‐mediated immunity.

Here, we demonstrate that *Angptl2* deficiency in tumor cells enhances the activation of CD8^+^ T cells in kidney tissues and slows tumor progression in a tRCC mouse model. Importantly, *Angptl2*‐deficient tRCC cells showed enhanced IFNγ‐induced expression of MHC‐I and antigen presentation machinery‐related genes. We also demonstrated that *Angptl2*‐deficient tRCC cells show enhanced intracellular MHC‐I antigen processing, leading to accelerated CD8^+^ T‐cell activation and tumor cell eradication. Mechanistically, we show that in tumor cells the ANGPTL2‐α5β1 integrin pathway promotes PRC2‐mediated H3K27me3 deposition at promoters of antigen presentation‐related genes, including MHC‐I, via JARID2 induction. These findings suggest that the ANGPTL2‐α5β1 integrin pathway attenuates IFNγ‐induced MHC‐I expression in tumor cells by accelerating PRC2‐mediated repressive histone modification, decreasing tumor cell susceptibility to CD8^+^ T‐cell‐mediated anti‐tumor immune responses (Fig. 7).

We previously demonstrated that ANGPTL2 has a dual function in tumor progression in both the tRCC mouse model and a murine syngeneic model [13]. Tumor stroma‐derived ANGPTL2 enhanced CD8^+^ T‐cell‐mediated anti‐tumor immune responses by activating dendritic cells via the PIR‐B receptor, leading to tumor suppression [13]. By contrast, tumor cell‐derived ANGPTL2 accelerated tumor progression [13]. Accordingly, here, we demonstrate that tumor cell‐derived ANGPTL2 contributes to tumor cell evasion of CD8^+^ T‐cell‐mediated anti‐tumor immunity by suppressing MHC‐I expression. Importantly, we found that α5β1 integrin, but not PIR‐B, is expressed on tumor cells and essential for ANGPTL2‐mediated epigenetic MHC‐I repression in tumor cells, suggesting that ANGPTL2 derived from tumor stroma or tumor cells acts through distinct receptors, namely PIR‐B or α5β1 integrin, respectively, to promote signaling with opposing effects on anti‐tumor immunity. Furthermore, flow cytometry analysis revealed that vascular cells (CD45^−^ EpCAM^−^ CD31^+^) express both α5 and β1 integrins, while other stromal cells (CD45^−^ EpCAM^−^ CD31^−^) express only β1 integrin (Fig. S6), suggesting that vascular cells could also be a target of ANGPTL2 signaling in the tRCC tumor microenvironment. In fact, we previously reported that ANGPTL2 promotes tumor angiogenesis in skin cancer and osteosarcoma [16, 40]. Thus, α5β1 integrin‐expressing cells, such as tumor and vascular cells, and PIR‐B‐expressing cells, such as dendritic cells, may compete for ANGPTL2 in the tumor microenvironment. Moreover, it remains unclear whether ANGPTL2 binding affinity for α5β1 integrin and PIR‐B differs; however, if ANGPTL2 binds more strongly to one of these receptors, relative abundance of ANGPTL2 in the tumor microenvironment may determine ANGPTL2 effects on anti‐tumor immunity.

Interestingly, although kidney tissues of CKO tRCC mice harbor ANGPTL2‐expressing stromal cells [13], those tissues show enhanced infiltration by and activation of CD8^+^ T cells relative to comparable tissues from tRCC mice. These observations support the idea that stroma‐derived ANGPTL2 does not function via integrin α5β1 or have a repressive effect on MHC‐I expression. Altered protein glycosylation is a hallmark of cancer and is reportedly associated with accelerated tumor growth, increased tumor cell invasivity, and evasion of immunosurveillance [41, 42]. ANGPTL2 is a glycoprotein [43], and integrins also are reportedly highly glycosylated type I membrane proteins [44]. Thus, changes in glycosylation of ANGPTL2 and/or its receptor in stromal versus tumor cells may also modulate ANGPTL2 activities in cancer pathology.

Although expression levels of *H2‐K1* and *H2‐D1* mRNAs in *Angptl2* KO and *Itga5* KO cells were equally increased relative to controls in the presence of IFNγ, the extent of increase in cell surface H‐2Db expression was markedly less than that of H‐2Kb, suggesting that H‐2Db expression is regulated post‐transcriptionally in tRCC cells. Indeed, others previously reported that RNA‐binding proteins can enhance or suppress translation of HLA class I proteins through binding to HLA class I mRNAs [45, 46], and thus, H‐2Db expression in tRCC cells may be similarly regulated.

Here, we demonstrate that *Angptl2* or *Itgα5* deficiency decreases *Jarid2* expression in tRCC cells. JARID2 not only recruits the PRC2 complex to target genes and but enhances EZH2 methyltransferase activity [34, 35, 36]. Importantly, we showed a significant decrease in H3K27me3 levels and EZH2 occupancy at promoters of antigen presentation‐related genes in *Angptl2* KO and *Itgα5* KO tRCC cells relative to controls, suggesting attenuation of JARID2‐mediated recruitment and/or activation of the PRC2 complex in these KO cells. However, it remains unclear how the ANGPTL2‐α5β1 integrin pathway regulates *JARID2* expression in tumor cells. A previous study demonstrated that tumor growth factor‐β (TGF‐β) upregulates *JARID2* expression in human lung and colon cancer cells [47]. *Jarid2* expression is also reportedly induced by nuclear factor of activated‐T cells (NFAT) in mouse immune cells [48]. Interestingly, we previously showed that ANGPTL2 enhances TGF‐β signaling in lung and skin cancers and in renal tubular epithelial cells [14, 40, 49]. Furthermore, we reported that ANGPTL2 activates NFATc3 in colon cancer cells [15]. Therefore, these pathways may regulate *JARID2* expression in tumor cells. Further studies are needed to define ANGPTL2‐mediated transcriptional regulatory mechanisms of *JARID2* in tumor cells.

A recent study reported that PRC2 complex containing MTF2 represses expression of *NLRC5* and its target genes, including MHC‐I and antigen presentation machinery‐related genes, in cancer cells [9]. However, here, we demonstrated that although *Angptl2* deficiency increases expression of MHC‐I and antigen presentation machinery‐related genes, it does not alter *Nlrc5* expression in tRCC cells. Furthermore, *Angptl2* KO tRCC cells showed decreased *Jarid2* expression, but not that of *Mtf2*, relative to control cells. The PRC2 complex can be classified as two subcomplexes, referred to as PRC2.1 and PRC2.2 [35, 36]. Both contain the same core subunits, while PRC2.1 and PRC2.2 contain MTF2 and JARID2 as respective non‐core subunits. Such non‐core subunits reportedly exhibit DNA‐binding properties and are essential for PRC2 complex recruitment to target genes [50, 51, 52]. Moreover, recent studies showed that PRC2.1 and PRC2.2 share most target genes, although some target genes are specific for each subcomplex [50, 51]. These findings suggest that antigen presentation‐related genes are common targets of both PRC2 subcomplexes in tumor cells, while *Nlrc5* repression may depend on PRC2.1, but not PRC2.2.

Cancer immunoediting is the process by which anti‐tumor immunity not only suppresses tumor development but contributes to tumor promotion [4, 7, 53]. In this process, tumor cell clones with different MHC‐I expression patterns are selected by T‐cell‐mediated anti‐tumor immunity, allowing MHC I‐negative or ‐low tumor cell clones to survive and preferentially form metastatic colonies by escaping anti‐tumor immune responses [4, 7, 53]. Interestingly, *ANGPTL2* mRNA levels show a significant positive correlation with those of *JARID2* mRNA, and levels of both are negatively correlated with those of *HLA‐B* and *HLA‐C* mRNAs in cancer lines derived from metastatic rather than primary lesions. These findings suggest that the ANGPTL2‐JARID2 axis in tumor cells could be associated with cancer immunoediting in various human cancers.

The PRC2 complex is implicated in silencing MHC‐I expression in small cell lung cancer (SCLC) and neuroblastoma but not in all cancer subtypes [9]. Together with results of analyses of TGCA datasets (Tables S5 and S6), these findings suggest that transcriptional repression mediated by the PRC2 complex may contribute to silencing of MHC‐I expression in certain types of cancer, such as ccRCC, SCLC, and neuroblastoma.

Although *HLA* class I gene mRNA levels were negatively correlated with *ANGPTL2* levels in ccRCC, we did not observe a correlation between *CD8A* and *ANGPTL2* mRNA levels. Tumor immune evasion can be facilitated not only by gene silencing or mutations in MHC‐I molecules and genes encoding IFNγ signaling pathway components in tumor cells, but also by changes in the tumor microenvironment [54, 55]. For example, tumor or tumor stromal cells, such as tumor‐associated macrophages, secrete chemokines and cytokines that can promote recruitment of immunosuppressive regulatory T or myeloid‐derived suppressor cells to the tumor microenvironment [54, 55]. Moreover, we previously reported that cancer‐associated fibroblast‐derived ANGPTL2 enhances CD8^+^ T‐cell‐mediated anti‐tumor immune responses [13]. Given the complexity of the tumor microenvironment and the fact that both tumor‐intrinsic and microenvironmental activities regulate anti‐tumor immunity, it may be challenging to correlate *ANGPTL2* mRNA levels with those of *CD8A*.

In this study, melanoma patients in the ANGPTL2‐low group showed shorter overall survival after treatment with PD‐1 inhibitors than patients in the ANGPTL2‐high group. ANGPTL2 is implicated in silencing MHC‐I expression in some types of cancer, but ANGPTL2 signaling may not repress MHC‐I expression in melanoma. In fact, we found that *ANGPTL2* mRNA levels were positively correlated with those of *HLA‐A*, *HLA‐B*, and *HLA‐C* in melanoma patients (Fig. S5B), suggesting that in this cancer context ANGPTL2 signaling does not regulate MHC‐I expression. Interestingly, a recent study reported that in patients with tRCC, the response rate to ICIs is greater than to tyrosine kinase inhibitors (TKIs) and that overall survival of tRCC patients treated with ICIs is prolonged relative to that of TKI‐treated patients [56]. Thus, ANGPTL2 may be a biomarker of unresponsiveness to ICI treatment or a therapeutic target for ICI resistance in tRCC. Further studies are needed to determine whether ANGPTL2 signaling in tumor cells contributes to acquisition of resistance to ICI therapy in both tRCC and ccRCC.

## 5 Conclusions

In conclusion, ANGPTL2 reduces tumor cell susceptibility to CD8^+^ T‐cell‐mediated anti‐tumor immune responses by promoting PRC2‐mediated repression of MHC‐I expression in tumor cells, thereby accelerating tumor progression. Our findings provide novel insight into mechanisms underlying tumor immune evasion and suggest that blocking ANGPTL2 signaling in tumor cells could be a potential strategy to promote tumor elimination by T‐cell‐mediated anti‐tumor immunity.

## Conflict of interest

The authors declare no conflict of interest.

## Author contributions

TsK and CH designed the study, performed and analyzed most experiments, and wrote the manuscript. TsK, CH, RK, HH, and SK collected mouse experimental data from the tRCC model. KM and KA generated *Angptl2* mutant mice. TsK, CH, RK, KM, KA, MB, and WML generated tRCC model mice. CH and RK established the mouse tRCC cell line R286. MB and WML established a HK‐2 cell line harboring the *PRCC‐TFE3* fusion gene and the human tRCC line UOK120. HK, SF, HH, and JM participated in statistical analysis and interpretation of results. ToK and YO coordinated, designed, and supervised the study, and wrote the manuscript. All authors discussed the data and commented on the manuscript.

### Peer review

The peer review history for this article is available at https://www.webofscience.com/api/gateway/wos/peer‐review/10.1002/1878‐0261.13490.

## Supporting information


**Fig. S1.** PRCC‐TFE3 proteins activate *ANGPTL2* transcription in tubular epithelial cells.
**Fig. S2.** The PRC2 complex contributes to repression of MHC‐I expression in tRCC cells.
**Fig. S3.** Expression of H‐2Kb, TAP1, and JARID2 protein in tumor lesions from kidney tissues derived from tRCC and CKO tRCC mice.
**Fig. S4.** Analysis of *ANGPTL2*, *ITGA5*, *JARID2*, *HLA* and *CD8A* mRNAs in human cancer cells.
**Fig. S5.** Correlation between *ANGPTL2* mRNA levels and overall survival after treatment with PD‐1 inhibitors in patients with metastatic melanoma.
**Fig. S6.** Expression of α5β1 integrin in vascular and stromal cells from kidney tissues of tRCC mice.

**Table S1.** sgRNAs used for CRISPR/Cas9‐mediated gene knockout.

**Table S2.** Primer pairs used for quantitative real time‐PCR.

**Table S3.** Primer pairs used for ChIP assay.

**Table S4.** A gene expression dataset of human cancer lines.

**Table S5.** A gene expression dataset of the ICGC/TCGA pan‐cancer analysis of the whole genomes consortium.

**Table S6.** A gene expression dataset of TGCA analysis of clear cell renal cell carcinoma.

**Table S7.** ANGPTL2 expression levels and overall survival of patients with metastatic melanoma treated with PD‐1 inhibitors.

## Data Availability

Data reported in this paper are available from the authors upon request. A gene expression dataset of human cancer lines was obtained from the public database Cancer Dependency Map (see Table S4). TGCA datasets were obtained from the public database cBioPortal for Cancer Genomics (see Tables [Link], [Link]).
