# Supplementary material for: ANGPTL2‐mediated epigenetic repression of MHC‐I in tumor cells accelerates tumor immune evasion
Source: Mol Oncol. 2023 Aug 7;17(12):2637–58. doi: 10.1002/1878-0261.13490 (PMC10701769; doi:10.1002/1878-0261.13490)
Supplement: Supplementary file 1 — Fig. S1. PRCC‐TFE3 proteins activate ANGPTL2 transcription in tubular epithelial cells. Fig. S2. The PRC2 complex contributes to repression of MHC‐I expression in tRCC cells. Fig. S3. Expression of H‐2Kb, TAP1, and JARID2 protein in tumor lesions from kidney tissues derived from tRCC and CKO tRCC mice. Fig. S4. Analysis of ANGPTL2, ITGA5, JARID2, HLA and CD8A mRNAs in human cancer cells. Fig. S5. Correlation between ANGPTL2 mRNA levels and overall survival after treatment with PD‐1 inhibitors in patients with metastatic melanoma. Fig. S6. Expression of α5β1 integrin in vascular and stromal cells from kidney tissues of tRCC mice. [file MOL2-17-2637-s003.pdf]

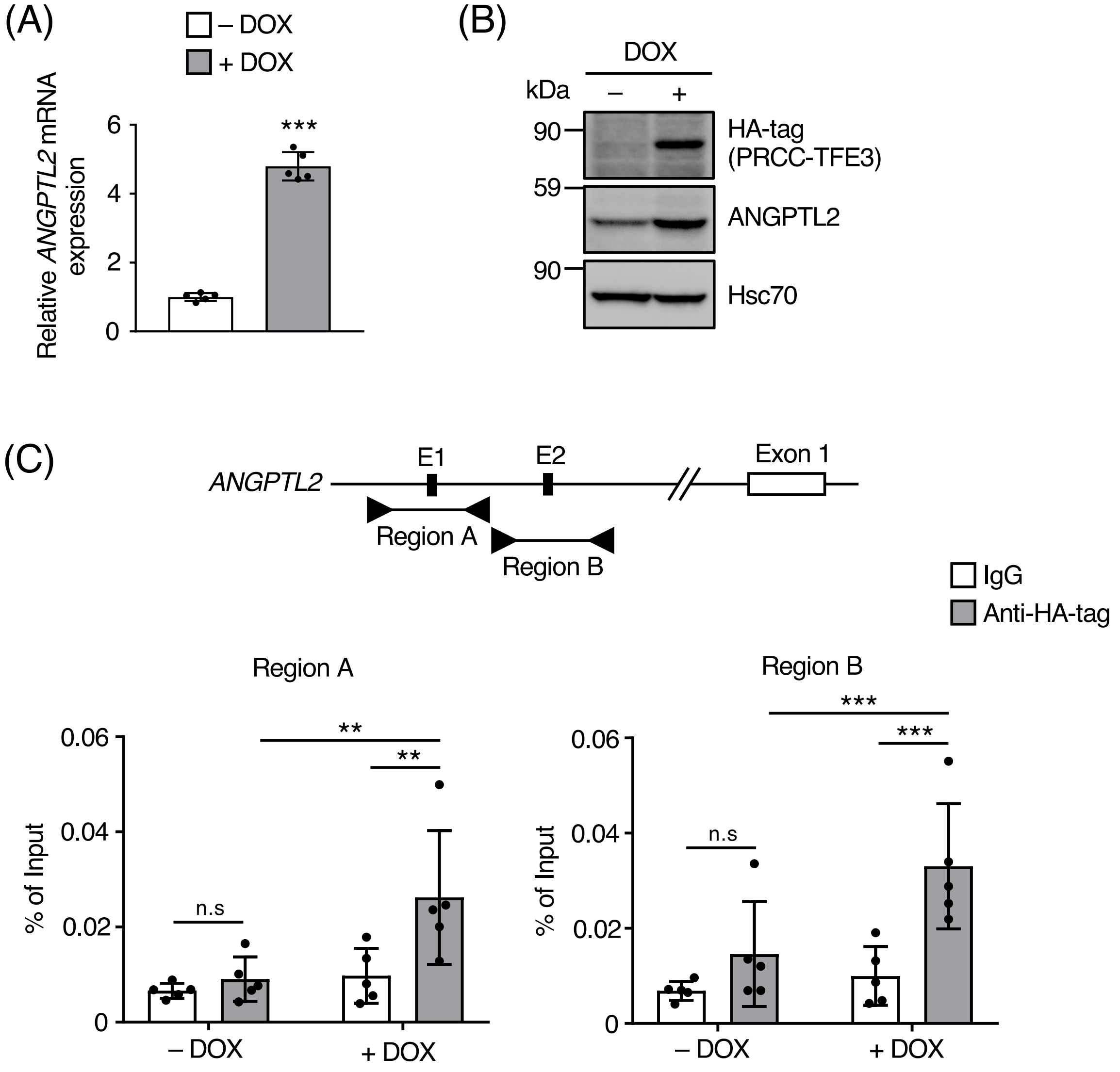

Kadomatsu *et al.*, Figure S1

Fig. S1. PRCC-TFE3 proteins activate *ANGPTL2* transcription in tubular epithelial cells. (A) Analysis of *ANGPTL2* expression in HK-2 cells harboring a doxycycline (DOX)-responsive, inducible HA-tagged PRCC-TFE3 gene. Shown are *ANGPTL2* mRNA levels in (DOX)-treated and untreated HK-2/PRCC-TFE3 cells (n = 5 per group). *ANGPTL2* levels in untreated cells were set to 1. Data are means ± SD. Statistical significance was determined by two-sided unpaired Student's *t*-test. ***p < 0.001. (B) Representative immunoblotting of HA-tagged PRCC-TFE3 and ANGPTL2 in (DOX)-treated and untreated HK-2/PRCC-TFE3 cells. Shown is a representative of two independent experiments. Hsc70 served as a loading control. (C) ChIP assay of HA-tagged PRCC-TFE3 at the *ANGPTL2* promoter region in (DOX)-treated and untreated HK-2/PRCC-TFE3 cells (n = 5 per group). Schematic showing E-box sites (E1 and E2) in the human ANGPTL2 promoter region (top) and PRCC-TFE3 occupancy at regions A and B corresponding to E-box sites. PRCC-TFE3 occupancy is shown as a percentage of input in graphs below. Data are means ± SD. Statistical significance was determined by two-way ANOVA with Tukey's post hoc test. **p < 0.01; ***p < 0.001; n.s, not significant.

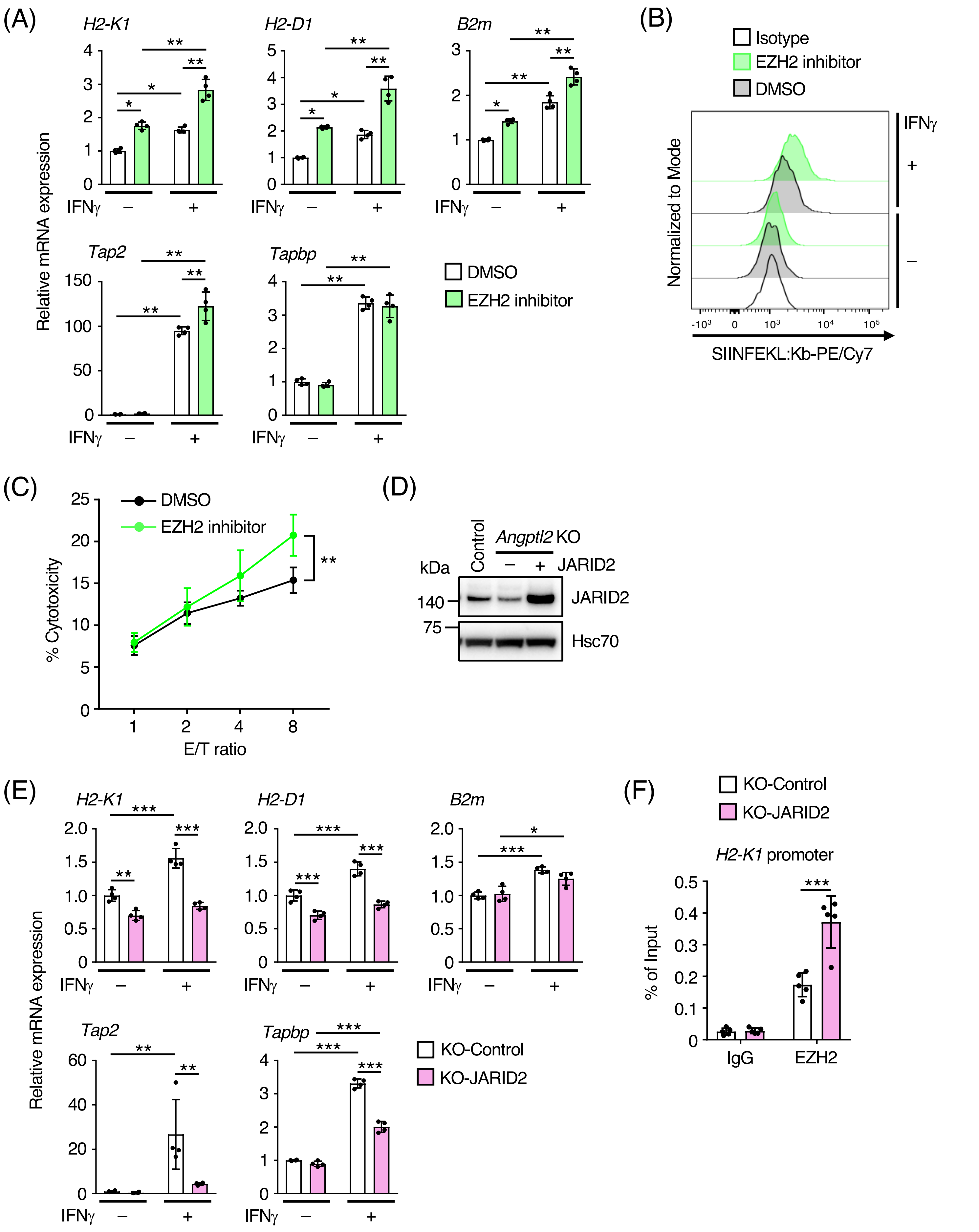

Kadomatsu *et al.*, Figure S2

Fig. S2. The PRC2 complex contributes to repression of MHC-I expression in tRCC cells. (A) Relative expression of mRNAs encoding MHC-I (*H2-K1* and *H2-D1*) and antigen presentation machinery-related factors (*B2m*, *Tap2*, and *Tapbp*) in EZH2 inhibitor-treated or untreated (DMSO) tRCC cells (n = 4 per group). Levels in untreated cells without IFNγ stimulation were set to 1. Data are means ± SD. Statistical significance was determined by two-way ANOVA with Tukey's post hoc test. *$p < 0.05$; **$p < 0.01$. (B) Representative histograms showing cell surface expression of SIINFEKL-bound H-2Kb in EZH2 inhibitor-treated or untreated (DMSO) tRCC-OVA cells in the presence or absence of IFNγ. Shown is a representative of three independent experiments. (C) T cell cytotoxicity assay with EZH2 inhibitor-treated or untreated (DMSO) tRCC cells (n = 4 per group). OT-I T cells were co-cultured 8 h with tumor cells at the indicated effector/target (E/T) ratios. Data are means ± SD. Statistical significance was determined by two-way ANOVA. **$p < 0.01$. (D) Representative immunoblotting of JARID2 in control KO cells and control (-) or JARID2-overexpressing (+) *Angptl2* KO cells. Shown is a representative of two independent experiments. Hsc70 served as a loading control. (E) Relative expression of mRNAs encoding MHC-I (*H2-K1* and *H2-D1*) and antigen presentation machinery-related factors (*B2m*, *Tap2*, and *Tapbp*) in control (KO-Control) and JARID2-overexpressing *Angptl2* KO (KO-JARID2) cells (n = 4 per group). Levels

in IFNγ-untreated KO-Control cells were set to 1. Data are means ± SD. Statistical significance was determined by two-way ANOVA with Tukey's post hoc test. *$p < 0.05$; **$p < 0.01$; ***$p < 0.001$. (F) ChIP assay for EZH2 at the *H2-K1* promoter in IFNγ-treated control (KO-Control) and JARID2-overexpressing *Angptl2* KO (KO-JARID2) cells (n = 5 per group). Data are means ± SD. EZH2 occupancy of the *H2-K1* promoter is presented as a percentage of input. Statistical significance was determined by two-way ANOVA with Sidak's post hoc test. ***$p < 0.001$.

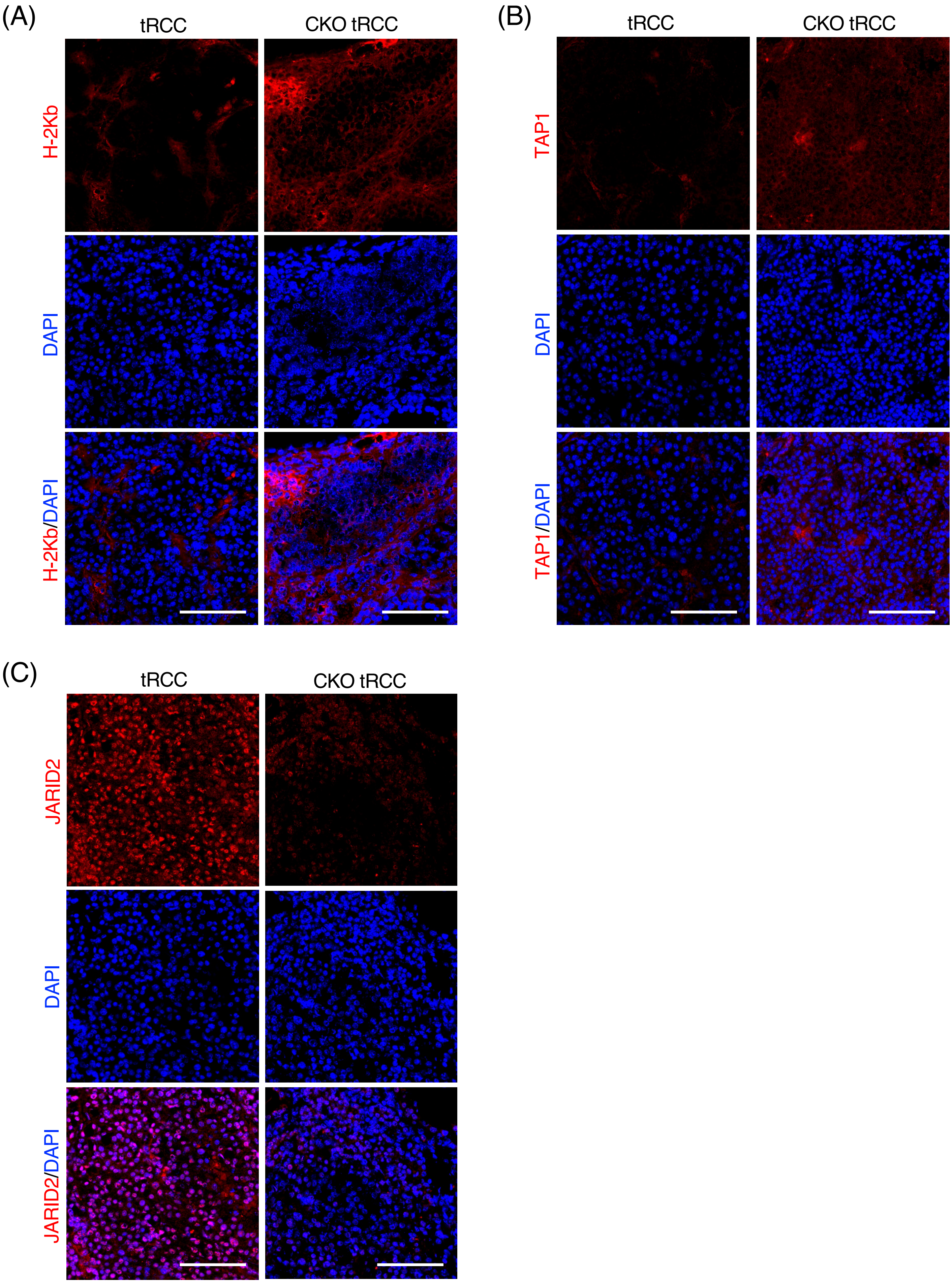

Kadomatsu *et al.*, Figure S3

Fig. S3. Expression of H-2Kb, TAP1, and JARID2 protein in tumor lesions from kidney tissues derived from tRCC and CKO tRCC mice. (A–C) Immunofluorescent staining for H-2Kb (A), TAP1 (B), and JARID2 (C) in tumor lesions from kidney tissues derived from tRCC and CKO tRCC mice at 45–50 weeks of age. Shown is a representative of two independent experiments. Nuclei are counterstained with DAPI. Scale bar, 100 μm.

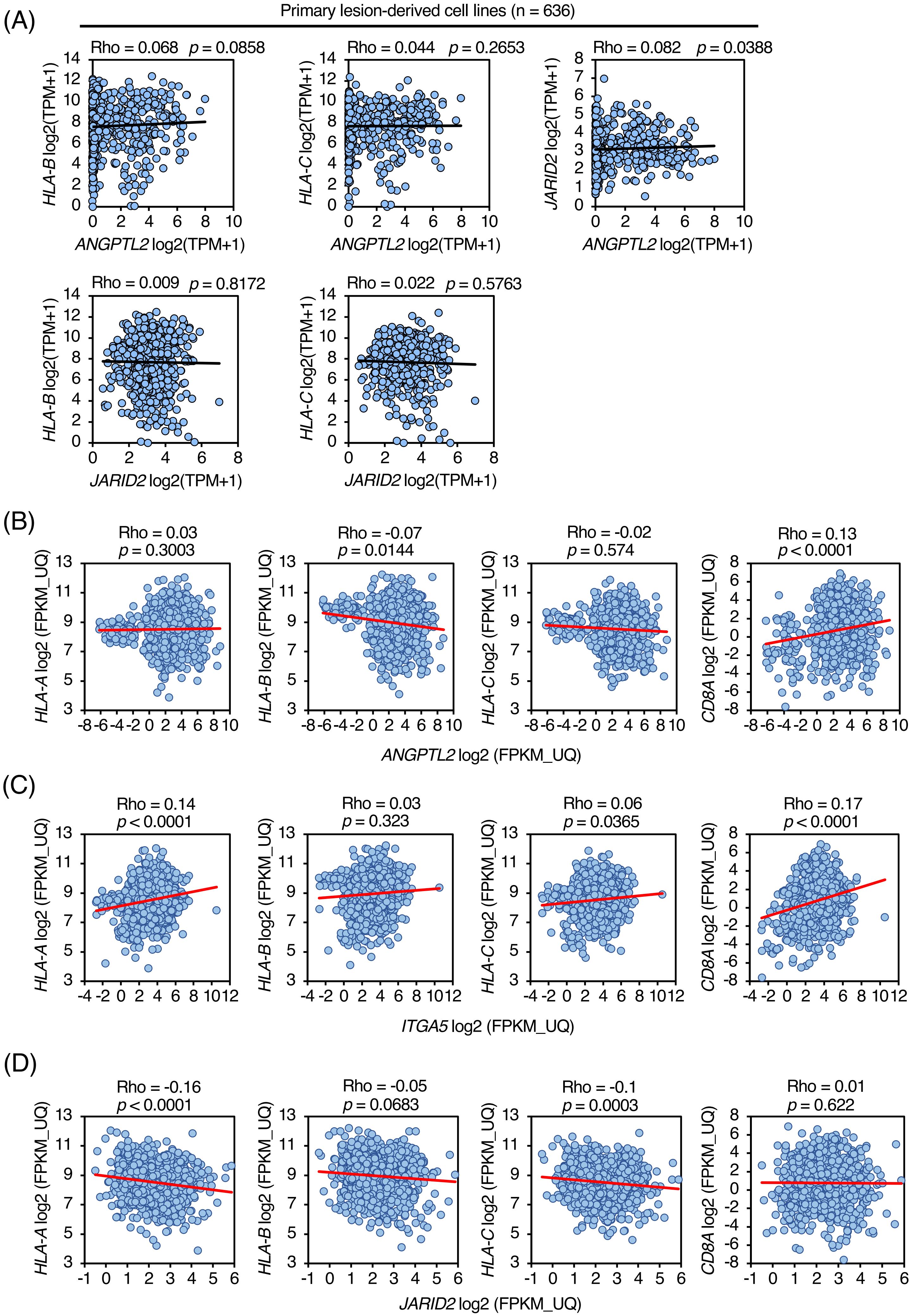

Kadomatsu *et al.*, Figure S4

Fig. S4. Analysis of *ANGPTL2*, *ITGA5*, *JARID2, HLA,* and *CD8A* mRNAs in human cancer cells. (A) Scatter plots showing correlations between expression levels of *ANGPTL2*, *HLA-B*, *HLA-C*, and *JARID2* mRNAs in primary lesion-derived cell lines. Shown is Spearman's correlation coefficient r (Rho). (B–D) Scatter plots showing correlations between expression levels of *ANGPTL2* (B), *ITGA5* (C), or *JARID2* (D) mRNAs and those of indicated genes in tumor samples (n = 1210). Shown is Spearman's correlation coefficient r (Rho).

(A)

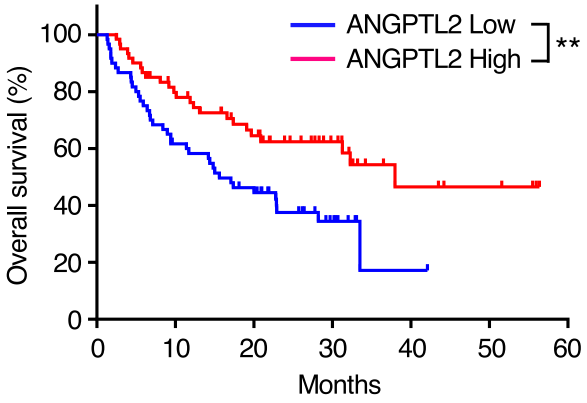

(B)

Correlations between expression levels of *ANGPTL2* and *HLA* class I mRNAs

Gene	Spearman's rho	*P* value
HLA-A	0.31	0.0006
HLA-B	0.3	0.0008
HLA-C	0.24	0.007

Kadomatsu *et al*., Figure S5

Fig. S5. Correlation between *ANGPTL2* mRNA levels and overall survival after treatment with PD-1 inhibitors in patients with metastatic melanoma. (A) Kaplan–Meier survival curves of patients in ANGPTL2 low (n = 60) and high (n = 61) groups. Statistical significance was determined by log-rank test. $^{**}p < 0.01$. (B) Correlations between expression levels of *ANGPTL2* and *HLA* class I mRNAs in pre-treatment biopsy samples (n = 121). Shown is Spearman's correlation coefficient r (Rho) and *P* values for correlations between *ANGPTL2* and *HLA* class I genes.

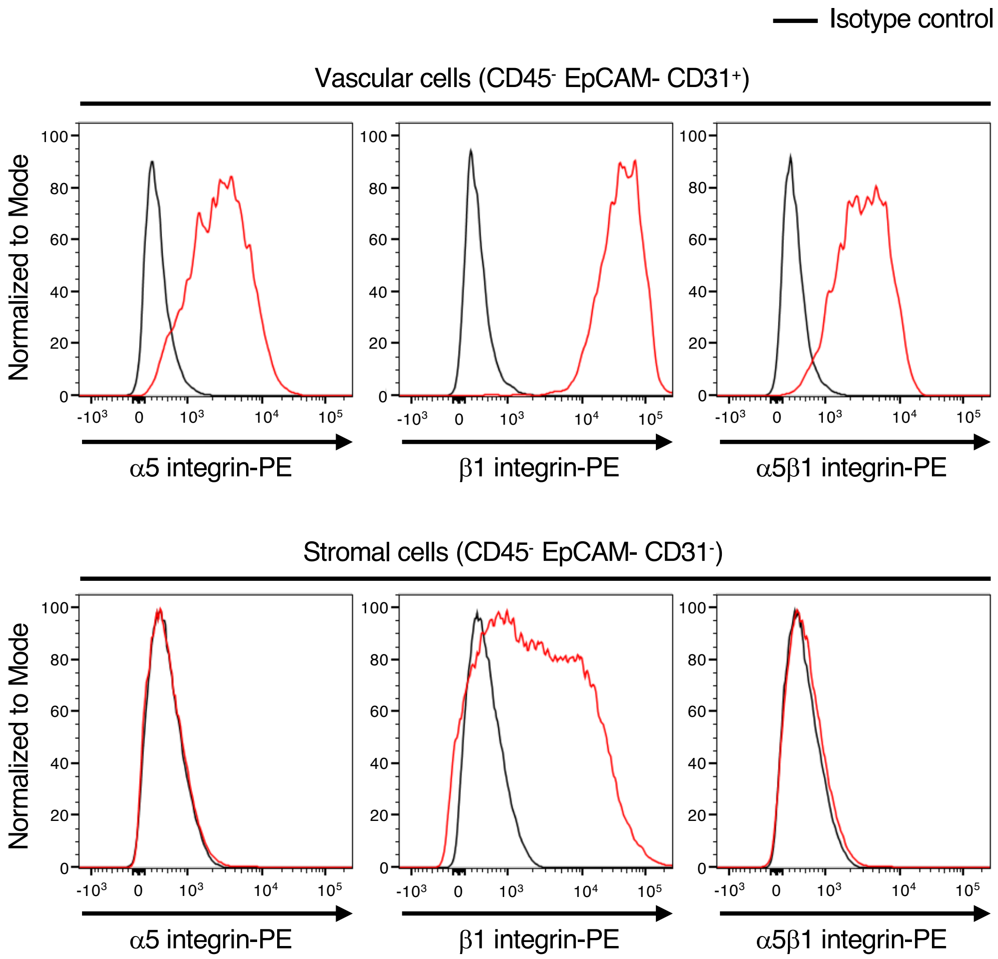

Kadomatsu *et al.*, Figure S6

Fig. S6. Expression of α5β1 integrin in vascular and stromal cells from kidney tissues of tRCC mice. Representative histograms showing cell surface expression of α5 integrin, β1 integrin, and α5β1 integrin in vascular ($CD45^-$ $EpCAM^-$ $CD31^+$) and other stromal ($CD45^-$ $EpCAM^-$ $CD31^+$) cells from kidney tissues of 52-week-old tRCC mice. Shown is a representative of two independent experiments.
