# Supplementary material for: ANGPTL2‐mediated epigenetic repression of MHC‐I in tumor cells accelerates tumor immune evasion
Source: Mol Oncol. 2023 Aug 7;17(12):2637–58. doi: 10.1002/1878-0261.13490 (PMC10701769; doi:10.1002/1878-0261.13490)
Supplement: Supplementary file 2 — Table S1. sgRNAs used for CRISPR/Cas9‐mediated gene knockout. [file MOL2-17-2637-s004.docx]

**Table S1. sgRNAs used for CRISPR/Cas9-mediated gene knockout**

| **Gene** | **Oligo** | **Sequences** |
| --- | --- | --- |
| *Angptl2* sgRNA1 | sense | CCGGTCGAAAGCGAGACAATGCGC |
|  | antisense | AAACGCGCATTGTCTCGCTTTCGA |
| *Angptl2* sgRNA2 | sense | CCGGGAATACCAACCGCCTGATGC |
|  | antisense | AAACGCATCAGGCGGTTGGTATTC |
| *Itgα5* sgRNA1 | sense | CCGGCCAGATCTCGTCCTCCTCGA |
|  | antisense | AAACTCGAGGAGGACGAGATCTGG |
| *Itgα5* sgRNA2 | sense | CCGGCAAGATGAGTTCAGCCGATT |
|  | antisense | AAACAATCGGCTGAACTCATCTTG |
