# Supplementary material for: ANGPTL2‐mediated epigenetic repression of MHC‐I in tumor cells accelerates tumor immune evasion
Source: Mol Oncol. 2023 Aug 7;17(12):2637–58. doi: 10.1002/1878-0261.13490 (PMC10701769; doi:10.1002/1878-0261.13490)
Supplement: Supplementary file 3 — Table S2. Primer pairs used for quantitative real time‐PCR. [file MOL2-17-2637-s007.docx]

**Table S2. Primer pairs used for quantitative real time-PCR**

| **Gene** |  | **Sequences** |
| --- | --- | --- |
| Mouse |  |  |
| *Rps18* | Forward | TTCTGGCCAACGGTCTAGACAAC |
|  | Reverse | CCAGTGGTCTTGGTGTGCTGA |
| *H2-K1* | Forward | CCGCAGATACCTGAAGAACG |
|  | Reverse | CAGCACCTCAGGGTGACTTTA |
| *H2-D1* | Forward | AGTGGTGCTGCAGAGCATTACAA |
|  | Reverse | GGTGACTTCACCTTTAGATCTGGG |
| *B2m* | Forward | TGGTGCTTGTCTCACTGACC |
|  | Reverse | TTCAGTATGTTCGGCTTCCC |
| *Tap2* | Forward | TGCCTGTTCTCTGTTGGGAG |
|  | Reverse | AGCTCCCCTGTCTTGGTCTC |
| *Tapbp* | Forward | GAGCCTGTCGTCATCACCAT |
|  | Reverse | ATCCTTTCCCAGCTGGACTC |
| *Irf1* | Forward | GGGACCCAGCTCTCTTCTGT |
|  | Reverse | AAAGCCAGCAAAAGACTCCC |
| *Nlrc5* | Forward | GCAGCTATGAGCCCTCCAAC |
|  | Reverse | CTGCTTAGAGAATTCATCTCTTGGG |
| *Ezh2* | Forward | AAGACAGCGACTGTGGTGCT |
|  | Reverse | ACAAACCGGTCCCTTCTCAG |
| *Eed* | Forward | CTGAAATTCCACCCACGAGA |
|  | Reverse | ACCTCCGAATATTGCCACAA |
| *Suz12* | Forward | TACATCGCCAACCTGGATTT |
|  | Reverse | AGAAACTCCGACATGCTTGC |
| *Mtf2* | Forward | TGCAAACGAAACTACCTCGG |
|  | Reverse | TTCTTCTCCGCAAATGTGGT |
| *Jarid2* | Forward | GAATGCACAGAAAAGGCAGC |
|  | Reverse | TCTGAGGCTGGTCCTAGTGC |
| Human |  |  |
| *ANGPTL2* | Forward | GCCACCAAGTGTCAGCCTCA |
|  | Reverse | TGGACAGTACCAAACATCCAACATC |
| *RPS18* | Forward | TTTGCGAGTACTCAACACCAACATC |
|  | Reverse | GAGCATATCTTCGGCCCACAC |
| *HLA-A* | Forward | GGCCCTGACCCAGACCTG |
|  | Reverse | GCACGAACTGCGTGTCGTC |
| *HLA-B* | Forward | CATCGTGGGCATTGTTGCTG |
|  | Reverse | ACGCAGCCTGAGAGTAGC |
| *HLA-C* | Forward | CTGGCCCTGACCGAGACCTG |
|  | Reverse | CGCTTGTACTTCTGTGTCTCC |
| *IRF1* | Forward | CAAATCCCGGGGCTCATCTGG |
|  | Reverse | CTGGCTCCTTTTCCCCTGCTTTGT |
| *NLRC5* | Forward | CACTTGGAGGAGCTGGACTT |
|  | Reverse | GTGAGTAAGCAAGGCCAAGG |
| *EZH2* | Forward | CGCTGACCATTGGGACAGTA |
|  | Reverse | TTGATAAAAATCCCCCAGCC |
| *EED* | Forward | CTGGTGGCAATATTTGGAGG |
|  | Reverse | GAATGATCCATACCACAGGACATT |
| *SUZ12* | Forward | AGTAGCCATGCAGGAAATGG |
|  | Reverse | ACTGCAACGTAGGTCCCTGA |
| *MTF2* | Forward | AAGGCAGAAAGGCATCCAA |
|  | Reverse | TTGTATATGGGCCAGGTGGA |
| *JARID2* | Forward | ATTGCTGGGAGCCTGAAAAC |
|  | Reverse | ACTTGGGATGCATCGTCCT |
