# Supplementary material for: ANGPTL2‐mediated epigenetic repression of MHC‐I in tumor cells accelerates tumor immune evasion
Source: Mol Oncol. 2023 Aug 7;17(12):2637–58. doi: 10.1002/1878-0261.13490 (PMC10701769; doi:10.1002/1878-0261.13490)
Supplement: Supplementary file 4 — Table S3. Primer pairs used for ChIP assay. [file MOL2-17-2637-s006.docx]

**Table S3. Primer pairs used for ChIP assay**

| **Gene** |  | **Sequences** |
| --- | --- | --- |
| *ANGPTL2* (Region A) | Forward | GGCTGGGATCATCTACAGGC |
|  | Reverse | AGAAGTCCAGCTAGAGGCCA |
| *ANGPTL2* (Region B) | Forward | GAACAGGGTAGAGACTGCGC |
|  | Reverse | CGAGAGGGTTTGTGACTTGC |
| *H2-K1* | Forward | GATCACCAAGAACCAATCAGTGTC |
|  | Reverse | ACGGTACCATCGCACCTGTC |
| *Tap1* | Forward | GTTTCTTCTTCCTCTAAACGCCAG |
|  | Reverse | GAGTCTCCGTGGGGAAGGAAG |
